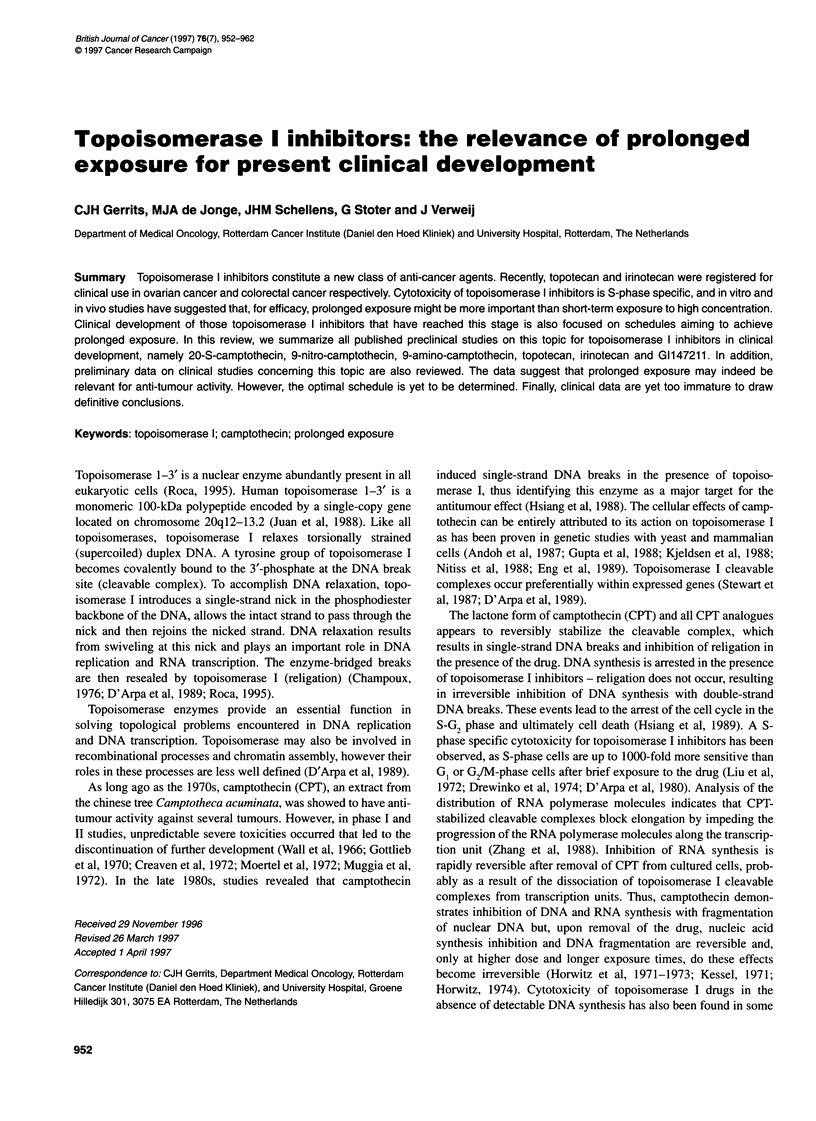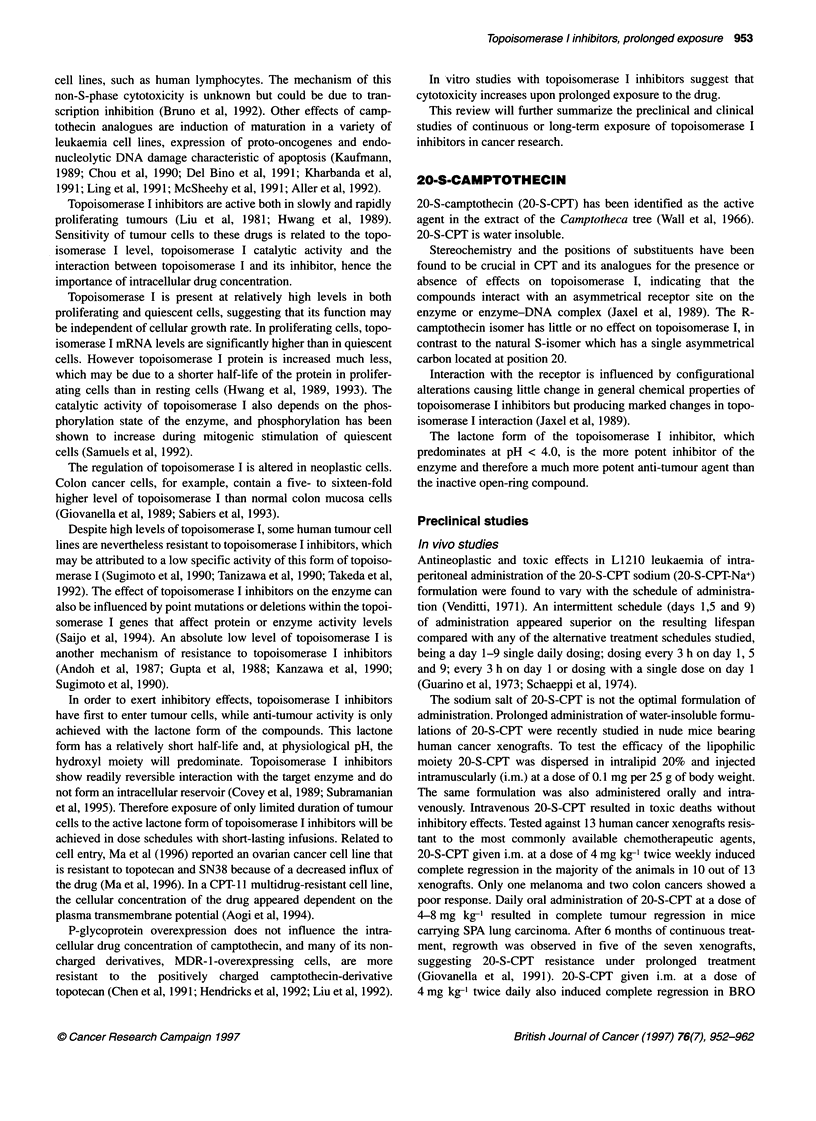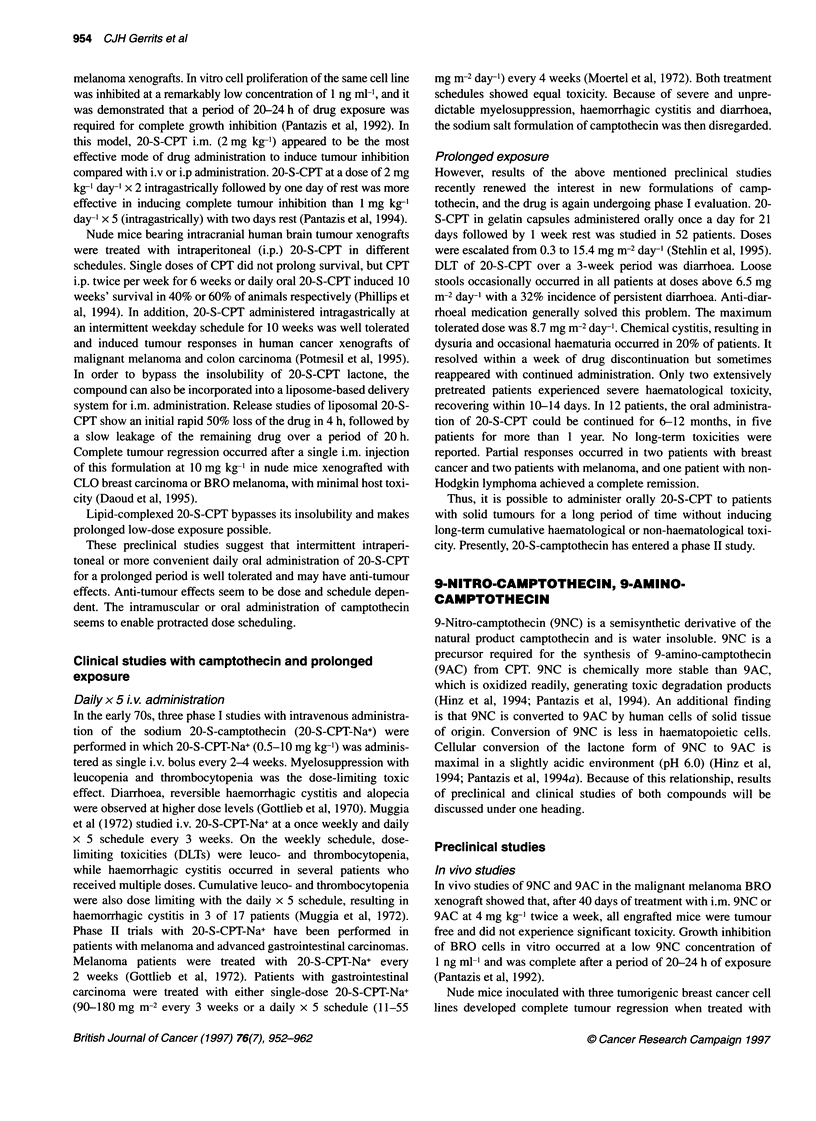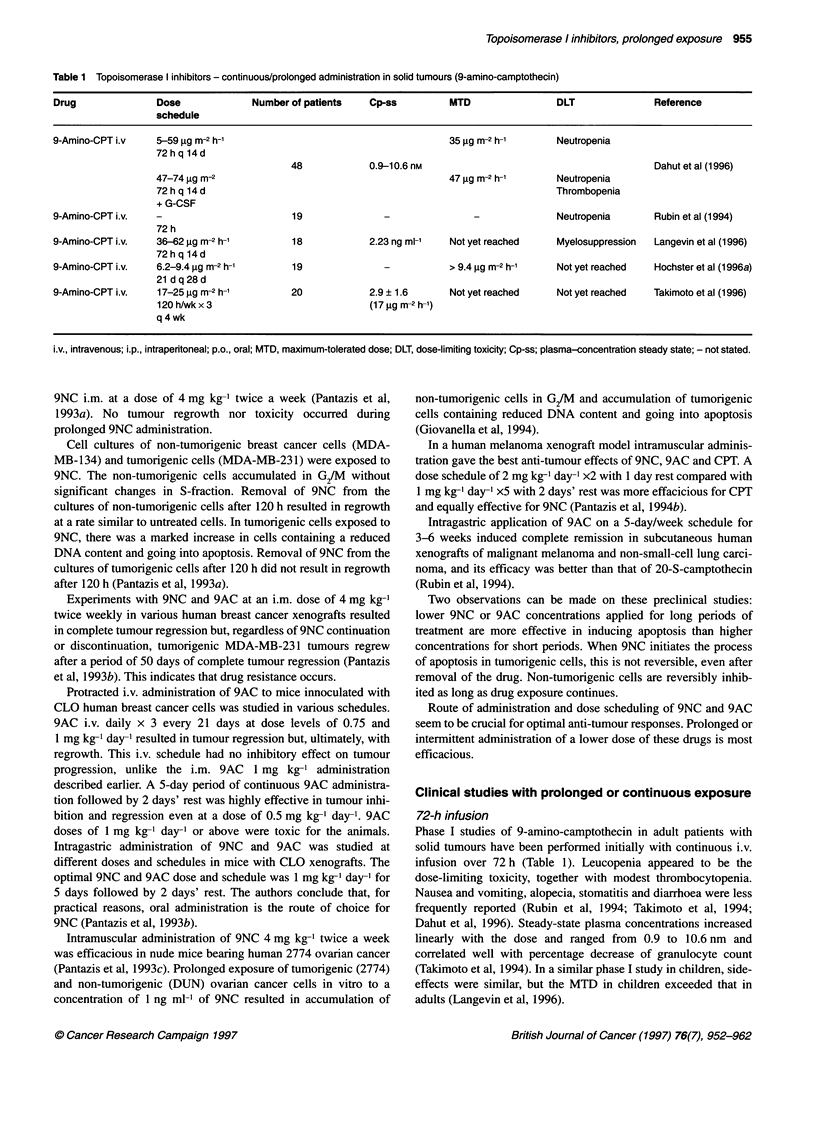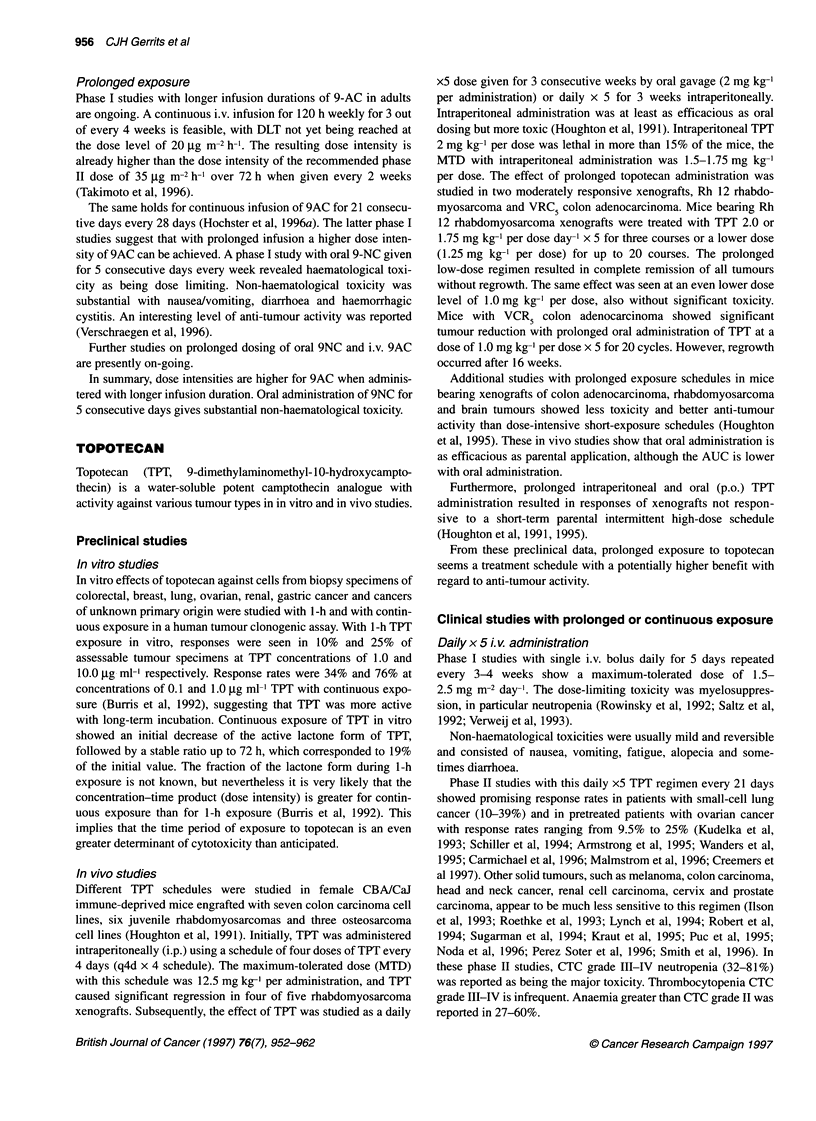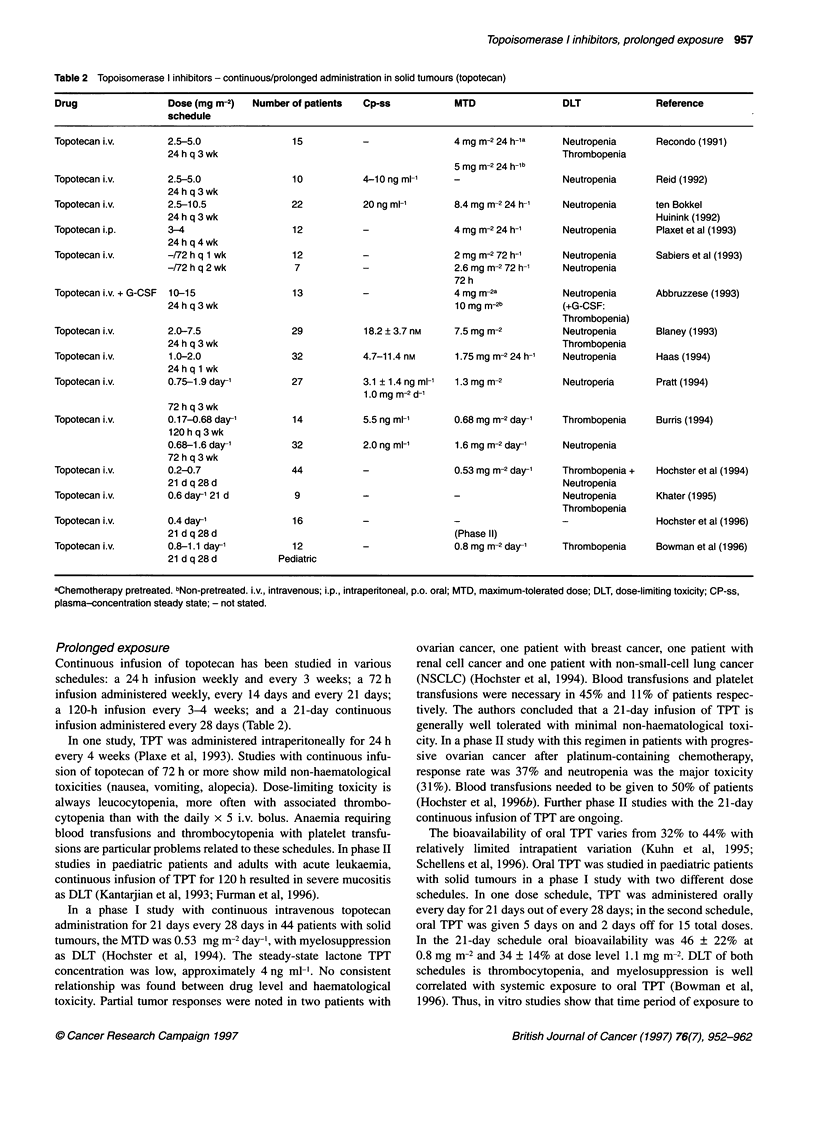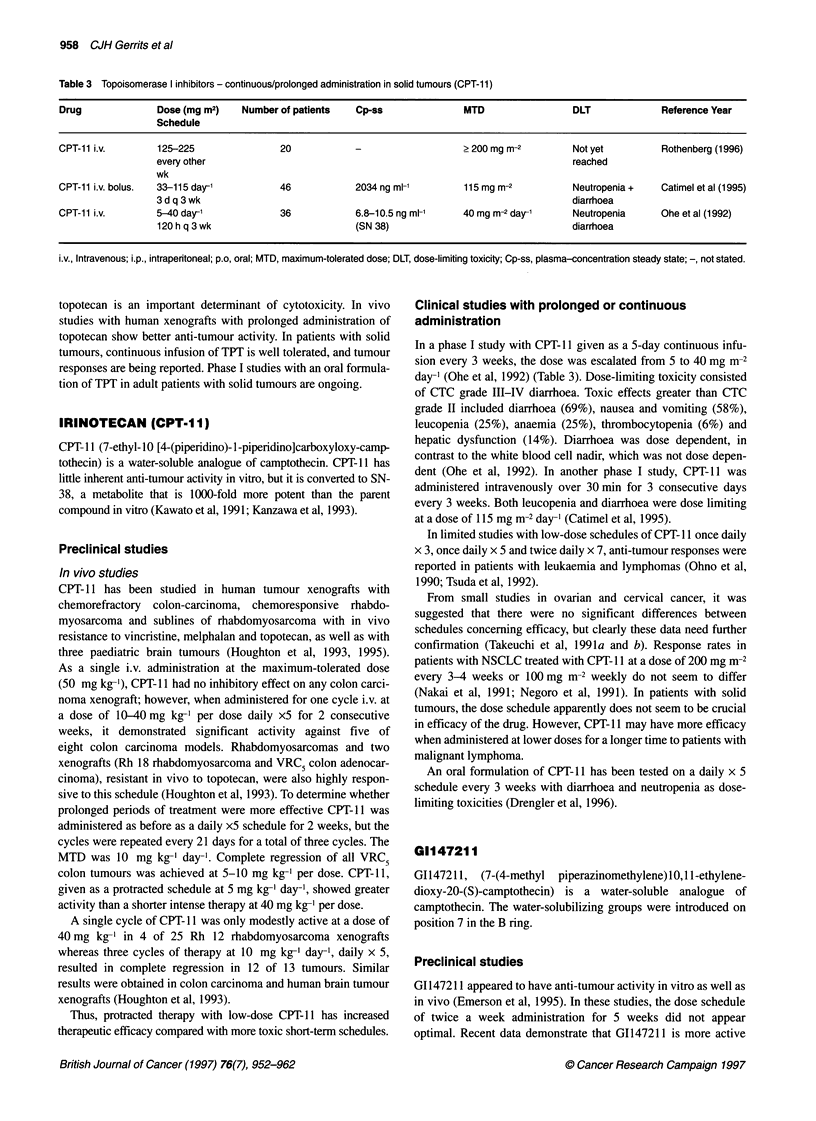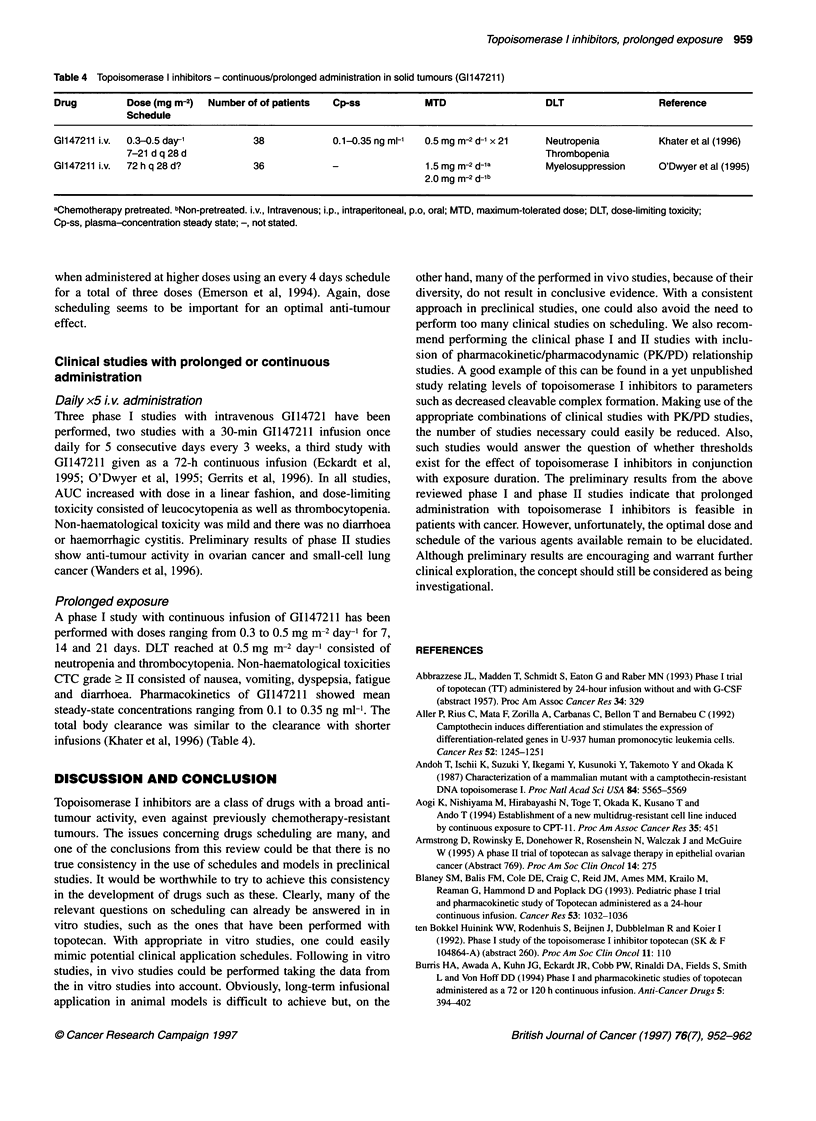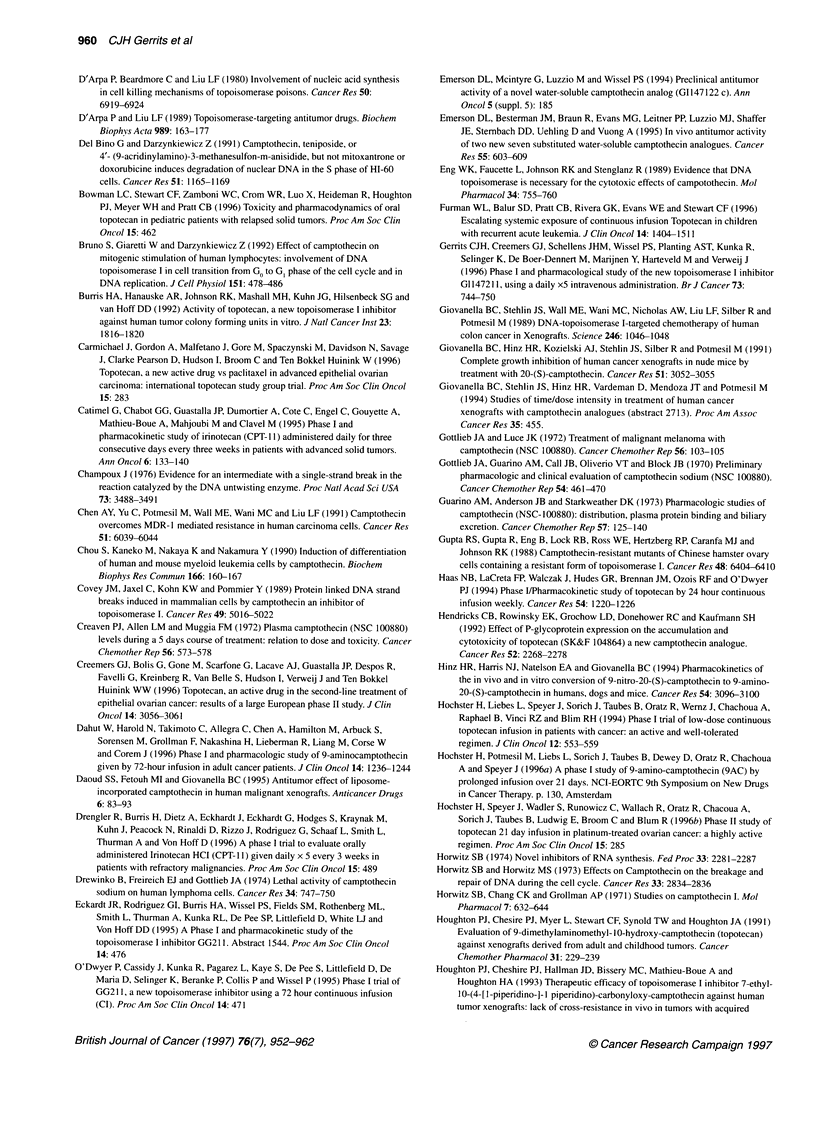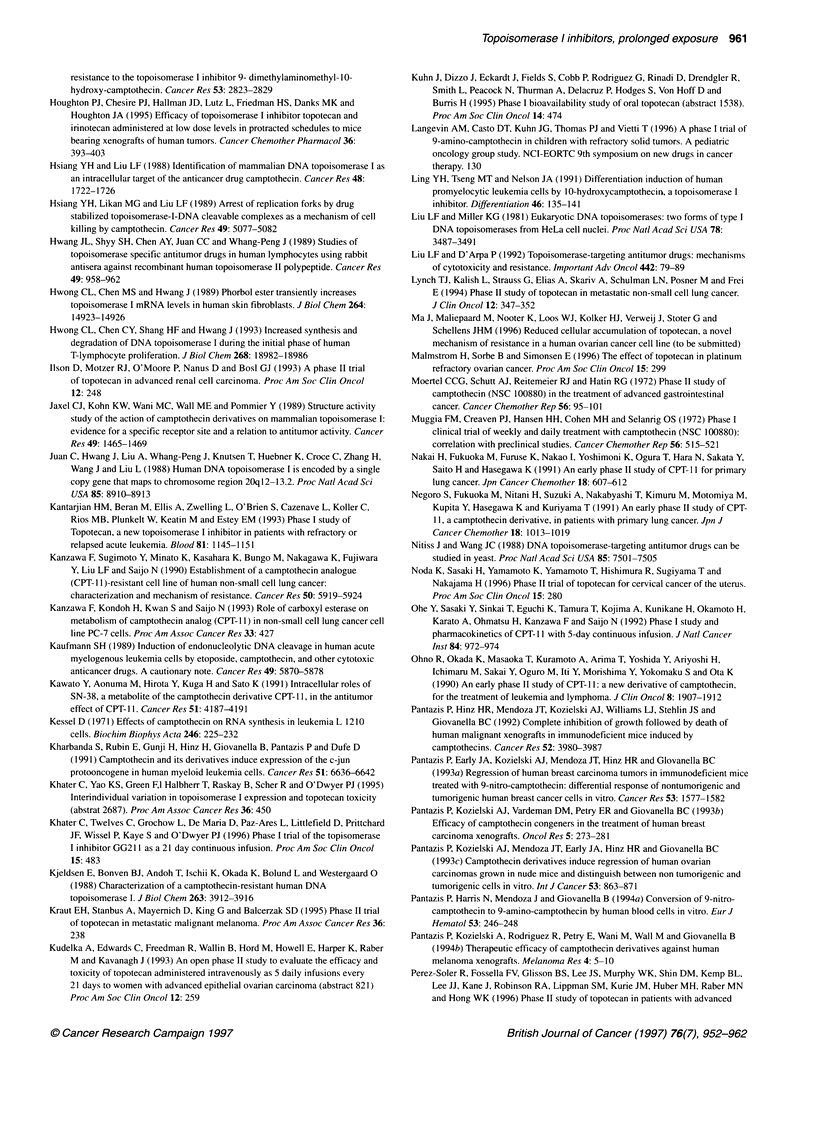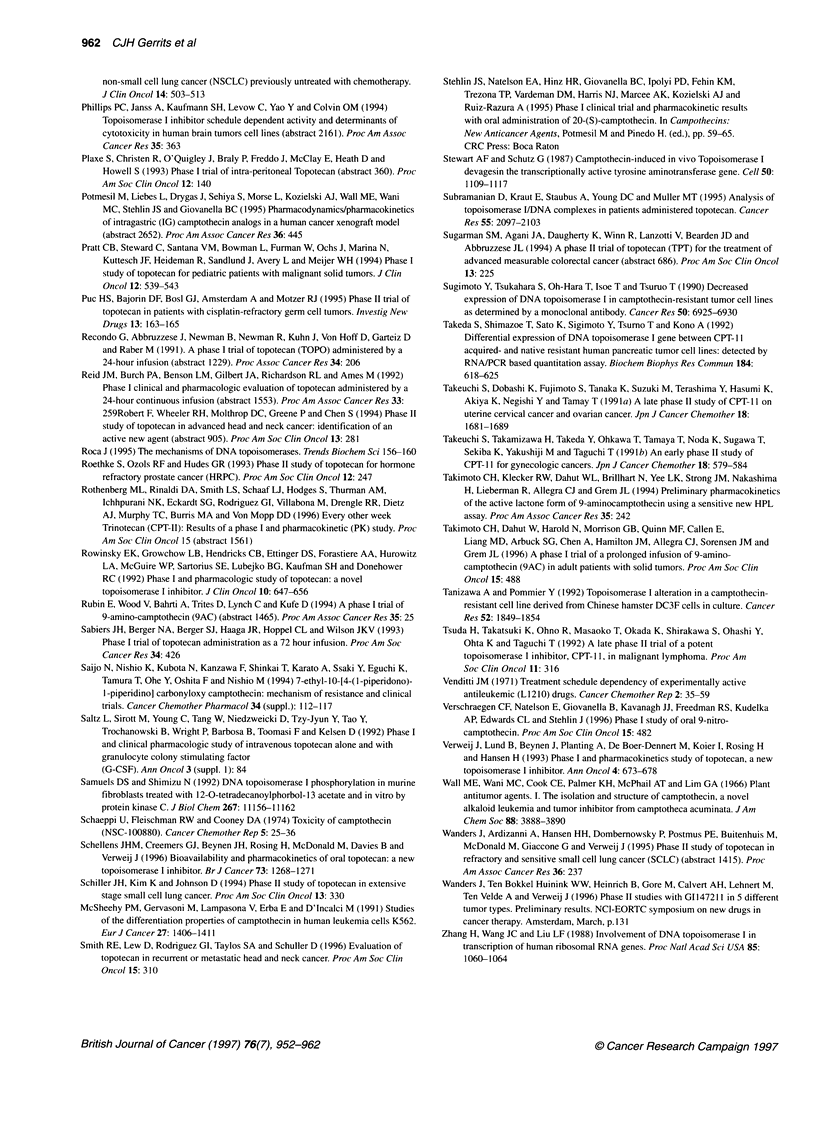# Topoisomerase I inhibitors: the relevance of prolonged exposure for present clinical development.

**DOI:** 10.1038/bjc.1997.491

**Published:** 1997

**Authors:** C. J. Gerrits, M. J. de Jonge, J. H. Schellens, G. Stoter, J. Verweij

**Affiliations:** Department of Medical Oncology, Rotterdam Cancer Institute (Daniel den Hoed Kliniek) and University Hospital, The Netherlands.

## Abstract

Topoisomerase I inhibitors constitute a new class of anti-cancer agents. Recently, topotecan and irinotecan were registered for clinical use in ovarian cancer and colorectal cancer respectively. Cytotoxicity of topoisomerase I inhibitors is S-phase specific, and in vitro and in vivo studies have suggested that, for efficacy, prolonged exposure might be more important than short-term exposure to high concentration. Clinical development of those topoisomerase I inhibitors that have reached this stage is also focused on schedules aiming to achieve prolonged exposure. In this review, we summarize all published preclinical studies on this topic for topoisomerase I inhibitors in clinical development, namely 20-S-camptothecin, 9-nitro-camptothecin, 9-amino-camptothecin, topotecan, irinotecan and GI147211. In addition, preliminary data on clinical studies concerning this topic are also reviewed. The data suggest that prolonged exposure may indeed be relevant for anti-tumour activity. However, the optimal schedule is yet to be determined. Finally, clinical data are yet too immature to draw definitive conclusions.


*British Journal of Cancer* (1997) **76**(7), 952–962
© 1997 Cancer Research Campaign

### Topoisomerase I inhibitors: the relevance of prolonged exposure for present clinical development

**CJH Gerrits, MJA de Jonge, JHM Schellens, G Stoter and J Verweij**

Department of Medical Oncology, Rotterdam Cancer Institute (Daniel den Hoed Kliniek) and University Hospital, Rotterdam, The Netherlands

**Summary** Topoisomerase I inhibitors constitute a new class of anti-cancer agents. Recently, topotecan and irinotecan were registered for clinical use in ovarian cancer and colorectal cancer respectively. Cytotoxicity of topoisomerase I inhibitors is S-phase specific, and in vitro and in vivo studies have suggested that, for efficacy, prolonged exposure might be more important than short-term exposure to high concentration. Clinical development of those topoisomerase I inhibitors that have reached this stage is also focused on schedules aiming to achieve prolonged exposure. In this review, we summarize all published preclinical studies on this topic for topoisomerase I inhibitors in clinical development, namely 20-S-camptothecin, 9-nitro-camptothecin, 9-amino-camptothecin, topotecan, irinotecan and GI147211. In addition, preliminary data on clinical studies concerning this topic are also reviewed. The data suggest that prolonged exposure may indeed be relevant for anti-tumour activity. However, the optimal schedule is yet to be determined. Finally, clinical data are yet too immature to draw definitive conclusions.

**Keywords**: topoisomerase I; camptothecin; prolonged exposure

Topoisomerase 1–3′ is a nuclear enzyme abundantly present in all eukaryotic cells (Roca, 1995). Human topoisomerase 1–3′ is a monomeric 100-kDa polypeptide encoded by a single-copy gene located on chromosome 20q12–13.2 (Juan et al, 1988). Like all topoisomerases, topoisomerase I relaxes torsionally strained (supercoiled) duplex DNA. A tyrosine group of topoisomerase I becomes covalently bound to the 3′-phosphate at the DNA break site (cleavable complex). To accomplish DNA relaxation, topoisomerase I introduces a single-strand nick in the phosphodiester backbone of the DNA, allows the intact strand to pass through the nick and then rejoins the nicked strand. DNA relaxation results from swiveling at this nick and plays an important role in DNA replication and RNA transcription. The enzyme-bridged breaks are then resealed by topoisomerase I (religation) (Champoux, 1976; D'Arpa et al, 1989; Roca, 1995).

Topoisomerase enzymes provide an essential function in solving topological problems encountered in DNA replication and DNA transcription. Topoisomerase may also be involved in recombinational processes and chromatin assembly, however their roles in these processes are less well defined (D′Arpa et al, 1989).

As long ago as the 1970s, camptothecin (CPT), an extract from the chinese tree *Camptotheca acuminata*, was showed to have anti-tumour activity against several tumours. However, in phase I and II studies, unpredictable severe toxicities occurred that led to the discontinuation of further development (Wall et al, 1966; Gottlieb et al, 1970; Creaven et al, 1972; Moertel et al, 1972; Muggia et al, 1972). In the late 1980s, studies revealed that camptothecin induced single-strand DNA breaks in the presence of topoisomerase I, thus identifying this enzyme as a major target for the antitumour effect (Hsiang et al, 1988). The cellular effects of camptothecin can be entirely attributed to its action on topoisomerase I as has been proven in genetic studies with yeast and mammalian cells (Andoh et al, 1987; Gupta et al, 1988; Kjeldsen et al, 1988; Nitiss et al, 1988; Eng et al, 1989). Topoisomerase I cleavable complexes occur preferentially within expressed genes (Stewart et al, 1987; D'Arpa et al, 1989).

The lactone form of camptothecin (CPT) and all CPT analogues appears to reversibly stabilize the cleavable complex, which results in single-strand DNA breaks and inhibition of religation in the presence of the drug. DNA synthesis is arrested in the presence of topoisomerase I inhibitors – religation does not occur, resulting in irreversible inhibition of DNA synthesis with double-strand DNA breaks. These events lead to the arrest of the cell cycle in the S-$G_2$ phase and ultimately cell death (Hsiang et al, 1989). A S-phase specific cytotoxicity for topoisomerase I inhibitors has been observed, as S-phase cells are up to 1000-fold more sensitive than $G_1$ or $G_2$/M-phase cells after brief exposure to the drug (Liu et al, 1972; Drewinko et al, 1974; D'Arpa et al, 1980). Analysis of the distribution of RNA polymerase molecules indicates that CPT-stabilized cleavable complexes block elongation by impeding the progression of the RNA polymerase molecules along the transcription unit (Zhang et al, 1988). Inhibition of RNA synthesis is rapidly reversible after removal of CPT from cultured cells, probably as a result of the dissociation of topoisomerase I cleavable complexes from transcription units. Thus, camptothecin demonstrates inhibition of DNA and RNA synthesis with fragmentation of nuclear DNA but, upon removal of the drug, nucleic acid synthesis inhibition and DNA fragmentation are reversible and, only at higher dose and longer exposure times, do these effects become irreversible (Horwitz et al, 1971–1973; Kessel, 1971; Horwitz, 1974). Cytotoxicity of topoisomerase I drugs in the absence of detectable DNA synthesis has also been found in some

*Received 29 November 1996*
*Revised 26 March 1997*
*Accepted 1 April 1997*

*Correspondence to:* CJH Gerrits, Department Medical Oncology, Rotterdam Cancer Institute (Daniel den Hoed Kliniek), and University Hospital, Groene Hilledijk 301, 3075 EA Rotterdam, The Netherlands cell lines, such as human lymphocytes. The mechanism of this non-S-phase cytotoxicity is unknown but could be due to transcription inhibition (Bruno et al, 1992). Other effects of camptothecin analogues are induction of maturation in a variety of leukaemia cell lines, expression of proto-oncogenes and endonucleolytic DNA damage characteristic of apoptosis (Kaufmann, 1989; Chou et al, 1990; Del Bino et al, 1991; Kharbanda et al, 1991; Ling et al, 1991; McSheehy et al, 1991; Aller et al, 1992).

Topoisomerase I inhibitors are active both in slowly and rapidly proliferating tumours (Liu et al, 1981; Hwang et al, 1989). Sensitivity of tumour cells to these drugs is related to the topoisomerase I level, topoisomerase I catalytic activity and the interaction between topoisomerase I and its inhibitor, hence the importance of intracellular drug concentration.

Topoisomerase I is present at relatively high levels in both proliferating and quiescent cells, suggesting that its function may be independent of cellular growth rate. In proliferating cells, topoisomerase I mRNA levels are significantly higher than in quiescent cells. However topoisomerase I protein is increased much less, which may be due to a shorter half-life of the protein in proliferating cells than in resting cells (Hwang et al, 1989, 1993). The catalytic activity of topoisomerase I also depends on the phosphorylation state of the enzyme, and phosphorylation has been shown to increase during mitogenic stimulation of quiescent cells (Samuels et al, 1992).

The regulation of topoisomerase I is altered in neoplastic cells. Colon cancer cells, for example, contain a five- to sixteen-fold higher level of topoisomerase I than normal colon mucosa cells (Giovanella et al, 1989; Sabiers et al, 1993).

Despite high levels of topoisomerase I, some human tumour cell lines are nevertheless resistant to topoisomerase I inhibitors, which may be attributed to a low specific activity of this form of topoisomerase I (Sugimoto et al, 1990; Tanizawa et al, 1990; Takeda et al, 1992). The effect of topoisomerase I inhibitors on the enzyme can also be influenced by point mutations or deletions within the topoisomerase I genes that affect protein or enzyme activity levels (Saijo et al, 1994). An absolute low level of topoisomerase I is another mechanism of resistance to topoisomerase I inhibitors (Andoh et al, 1987; Gupta et al, 1988; Kanzawa et al, 1990; Sugimoto et al, 1990).

In order to exert inhibitory effects, topoisomerase I inhibitors have first to enter tumour cells, while anti-tumour activity is only achieved with the lactone form of the compounds. This lactone form has a relatively short half-life and, at physiological pH, the hydroxyl moiety will predominate. Topoisomerase I inhibitors show readily reversible interaction with the target enzyme and do not form an intracellular reservoir (Covey et al, 1989; Subramanian et al, 1995). Therefore exposure of only limited duration of tumour cells to the active lactone form of topoisomerase I inhibitors will be achieved in dose schedules with short-lasting infusions. Related to cell entry, Ma et al (1996) reported an ovarian cancer cell line that is resistant to topotecan and SN38 because of a decreased influx of the drug (Ma et al, 1996). In a CPT-11 multidrug-resistant cell line, the cellular concentration of the drug appeared dependent on the plasma transmembrane potential (Aogi et al, 1994).

P-glycoprotein overexpression does not influence the intracellular drug concentration of camptothecin, and many of its non-charged derivatives, MDR-1-overexpressing cells, are more resistant to the positively charged camptothecin-derivative topotecan (Chen et al, 1991; Hendricks et al, 1992; Liu et al, 1992).

In vitro studies with topoisomerase I inhibitors suggest that cytotoxicity increases upon prolonged exposure to the drug.

This review will further summarize the preclinical and clinical studies of continuous or long-term exposure of topoisomerase I inhibitors in cancer research.

### 20-S-CAMPTOTHECIN

20-S-camptothecin (20-S-CPT) has been identified as the active agent in the extract of the *Camptotheca* tree (Wall et al, 1966). 20-S-CPT is water insoluble.

Stereochemistry and the positions of substituents have been found to be crucial in CPT and its analogues for the presence or absence of effects on topoisomerase I, indicating that the compounds interact with an asymmetrical receptor site on the enzyme or enzyme–DNA complex (Jaxel et al, 1989). The R-camptothecin isomer has little or no effect on topoisomerase I, in contrast to the natural S-isomer which has a single asymmetrical carbon located at position 20.

Interaction with the receptor is influenced by configurational alterations causing little change in general chemical properties of topoisomerase I inhibitors but producing marked changes in topoisomerase I interaction (Jaxel et al, 1989).

The lactone form of the topoisomerase I inhibitor, which predominates at pH < 4.0, is the more potent inhibitor of the enzyme and therefore a much more potent anti-tumour agent than the inactive open-ring compound.

### Preclinical studies

#### *In vivo studies*

Antineoplastic and toxic effects in L1210 leukaemia of intraperitoneal administration of the 20-S-CPT sodium (20-S-CPT-$Na^+$) formulation were found to vary with the schedule of administration (Venditti, 1971). An intermittent schedule (days 1,5 and 9) of administration appeared superior on the resulting lifespan compared with any of the alternative treatment schedules studied, being a day 1–9 single daily dosing; dosing every 3 h on day 1, 5 and 9; every 3 h on day 1 or dosing with a single dose on day 1 (Guarino et al, 1973; Schaeppi et al, 1974).

The sodium salt of 20-S-CPT is not the optimal formulation of administration. Prolonged administration of water-insoluble formulations of 20-S-CPT were recently studied in nude mice bearing human cancer xenografts. To test the efficacy of the lipophilic moiety 20-S-CPT was dispersed in intralipid 20% and injected intramuscularly (i.m.) at a dose of 0.1 mg per 25 g of body weight. The same formulation was also administered orally and intravenously. Intravenous 20-S-CPT resulted in toxic deaths without inhibitory effects. Tested against 13 human cancer xenografts resistant to the most commonly available chemotherapeutic agents, 20-S-CPT given i.m. at a dose of 4 mg $kg^{-1}$ twice weekly induced complete regression in the majority of the animals in 10 out of 13 xenografts. Only one melanoma and two colon cancers showed a poor response. Daily oral administration of 20-S-CPT at a dose of 4–8 mg $kg^{-1}$ resulted in complete tumour regression in mice carrying SPA lung carcinoma. After 6 months of continuous treatment, regrowth was observed in five of the seven xenografts, suggesting 20-S-CPT resistance under prolonged treatment (Giovanella et al, 1991). 20-S-CPT given i.m. at a dose of 4 mg $kg^{-1}$ twice daily also induced complete regression in BRO

© Cancer Research Campaign 1997

melanoma xenografts. In vitro cell proliferation of the same cell line was inhibited at a remarkably low concentration of 1 ng $ml^{-1}$, and it was demonstrated that a period of 20–24 h of drug exposure was required for complete growth inhibition (Pantazis et al, 1992). In this model, 20-S-CPT i.m. (2 mg $kg^{-1}$) appeared to be the most effective mode of drug administration to induce tumour inhibition compared with i.v or i.p administration. 20-S-CPT at a dose of 2 mg $kg^{-1}$ $day^{-1}$ × 2 intragastrically followed by one day of rest was more effective in inducing complete tumour inhibition than 1 mg $kg^{-1}$ $day^{-1}$ × 5 (intragastrically) with two days rest (Pantazis et al, 1994).

Nude mice bearing intracranial human brain tumour xenografts were treated with intraperitoneal (i.p.) 20-S-CPT in different schedules. Single doses of CPT did not prolong survival, but CPT i.p. twice per week for 6 weeks or daily oral 20-S-CPT induced 10 weeks' survival in 40% or 60% of animals respectively (Phillips et al, 1994). In addition, 20-S-CPT administered intragastrically at an intermittent weekday schedule for 10 weeks was well tolerated and induced tumour responses in human cancer xenografts of malignant melanoma and colon carcinoma (Potmesil et al, 1995). In order to bypass the insolubility of 20-S-CPT lactone, the compound can also be incorporated into a liposome-based delivery system for i.m. administration. Release studies of liposomal 20-S-CPT show an initial rapid 50% loss of the drug in 4 h, followed by a slow leakage of the remaining drug over a period of 20 h. Complete tumour regression occurred after a single i.m. injection of this formulation at 10 mg $kg^{-1}$ in nude mice xenografted with CLO breast carcinoma or BRO melanoma, with minimal host toxicity (Daoud et al, 1995).

Lipid-complexed 20-S-CPT bypasses its insolubility and makes prolonged low-dose exposure possible.

These preclinical studies suggest that intermittent intraperitoneal or more convenient daily oral administration of 20-S-CPT for a prolonged period is well tolerated and may have anti-tumour effects. Anti-tumour effects seem to be dose and schedule dependent. The intramuscular or oral administration of camptothecin seems to enable protracted dose scheduling.

### Clinical studies with camptothecin and prolonged exposure

### *Daily × 5 i.v. administration*

In the early 70s, three phase I studies with intravenous administration of the sodium 20-S-camptothecin (20-S-CPT-$Na^+$) were performed in which 20-S-CPT-$Na^+$ (0.5–10 mg $kg^{-1}$) was administered as single i.v. bolus every 2–4 weeks. Myelosuppression with leucopenia and thrombocytopenia was the dose-limiting toxic effect. Diarrhoea, reversible haemorrhagic cystitis and alopecia were observed at higher dose levels (Gottlieb et al, 1970). Muggia et al (1972) studied i.v. 20-S-CPT-$Na^+$ at a once weekly and daily × 5 schedule every 3 weeks. On the weekly schedule, dose-limiting toxicities (DLTs) were leuco- and thrombocytopenia, while haemorrhagic cystitis occurred in several patients who received multiple doses. Cumulative leuco- and thrombocytopenia were also dose limiting with the daily × 5 schedule, resulting in haemorrhagic cystitis in 3 of 17 patients (Muggia et al, 1972). Phase II trials with 20-S-CPT-$Na^+$ have been performed in patients with melanoma and advanced gastrointestinal carcinomas. Melanoma patients were treated with 20-S-CPT-$Na^+$ every 2 weeks (Gottlieb et al, 1972). Patients with gastrointestinal carcinoma were treated with either single-dose 20-S-CPT-$Na^+$ (90–180 mg $m^{-2}$ every 3 weeks or a daily × 5 schedule (11–55 mg $m^{-2}$ $day^{-1}$) every 4 weeks (Moertel et al, 1972). Both treatment schedules showed equal toxicity. Because of severe and unpredictable myelosuppression, haemorrhagic cystitis and diarrhoea, the sodium salt formulation of camptothecin was then disregarded.

### *Prolonged exposure*

However, results of the above mentioned preclinical studies recently renewed the interest in new formulations of camptothecin, and the drug is again undergoing phase I evaluation. 20-S-CPT in gelatin capsules administered orally once a day for 21 days followed by 1 week rest was studied in 52 patients. Doses were escalated from 0.3 to 15.4 mg $m^{-2}$ $day^{-1}$ (Stehlin et al, 1995). DLT of 20-S-CPT over a 3-week period was diarrhoea. Loose stools occasionally occurred in all patients at doses above 6.5 mg $m^{-2}$ $day^{-1}$ with a 32% incidence of persistent diarrhoea. Anti-diarrhoeal medication generally solved this problem. The maximum tolerated dose was 8.7 mg $m^{-2}$ $day^{-1}$. Chemical cystitis, resulting in dysuria and occasional haematuria occurred in 20% of patients. It resolved within a week of drug discontinuation but sometimes reappeared with continued administration. Only two extensively pretreated patients experienced severe haematological toxicity, recovering within 10–14 days. In 12 patients, the oral administration of 20-S-CPT could be continued for 6–12 months, in five patients for more than 1 year. No long-term toxicities were reported. Partial responses occurred in two patients with breast cancer and two patients with melanoma, and one patient with non-Hodgkin lymphoma achieved a complete remission.

Thus, it is possible to administer orally 20-S-CPT to patients with solid tumours for a long period of time without inducing long-term cumulative haematological or non-haematological toxicity. Presently, 20-S-camptothecin has entered a phase II study.

### 9-NITRO-CAMPTOTHECIN, 9-AMINO-CAMPTOTHECIN

9-Nitro-camptothecin (9NC) is a semisynthetic derivative of the natural product camptothecin and is water insoluble. 9NC is a precursor required for the synthesis of 9-amino-camptothecin (9AC) from CPT. 9NC is chemically more stable than 9AC, which is oxidized readily, generating toxic degradation products (Hinz et al, 1994; Pantazis et al, 1994). An additional finding is that 9NC is converted to 9AC by human cells of solid tissue of origin. Conversion of 9NC is less in haematopoietic cells. Cellular conversion of the lactone form of 9NC to 9AC is maximal in a slightly acidic environment (pH 6.0) (Hinz et al, 1994; Pantazis et al, 1994*a*). Because of this relationship, results of preclinical and clinical studies of both compounds will be discussed under one heading.

### Preclinical studies

### *In vivo studies*

In vivo studies of 9NC and 9AC in the malignant melanoma BRO xenograft showed that, after 40 days of treatment with i.m. 9NC or 9AC at 4 mg $kg^{-1}$ twice a week, all engrafted mice were tumour free and did not experience significant toxicity. Growth inhibition of BRO cells in vitro occurred at a low 9NC concentration of 1 ng $ml^{-1}$ and was complete after a period of 20–24 h of exposure (Pantazis et al, 1992).

Nude mice inoculated with three tumorigenic breast cancer cell lines developed complete tumour regression when treated with

 © Cancer Research Campaign 1997

**Table 1** Topoisomerase I inhibitors – continuous/prolonged administration in solid tumours (9-amino-camptothecin)

| Drug | Dose schedule | Number of patients | Cp-ss | MTD | DLT | Reference |
|---|---|---|---|---|---|---|
| 9-Amino-CPT i.v | 5–59 μg $m^{-2}$ $h^{-1}$ 72 h q 14 d | | | 35 μg $m^{-2}$ $h^{-1}$ | Neutropenia | |
| | | 48 | 0.9–10.6 nM | | | Dahut et al (1996) |
| | 47–74 μg $m^{-2}$ 72 h q 14 d + G-CSF | | | 47 μg $m^{-2}$ $h^{-1}$ | Neutropenia Thrombopenia | |
| 9-Amino-CPT i.v. | – 72 h | 19 | – | – | Neutropenia | Rubin et al (1994) |
| 9-Amino-CPT i.v. | 36–62 μg $m^{-2}$ $h^{-1}$ 72 h q 14 d | 18 | 2.23 ng $ml^{-1}$ | Not yet reached | Myelosuppression | Langevin et al (1996) |
| 9-Amino-CPT i.v. | 6.2–9.4 μg $m^{-2}$ $h^{-1}$ 21 d q 28 d | 19 | – | > 9.4 μg $m^{-2}$ $h^{-1}$ | Not yet reached | Hochster et al (1996*a*) |
| 9-Amino-CPT i.v. | 17–25 μg $m^{-2}$ $h^{-1}$ 120 h/wk × 3 q 4 wk | 20 | 2.9 ± 1.6 (17 μg $m^{-2}$ $h^{-1}$) | Not yet reached | Not yet reached | Takimoto et al (1996) |

i.v., intravenous; i.p., intraperitoneal; p.o., oral; MTD, maximum-tolerated dose; DLT, dose-limiting toxicity; Cp-ss; plasma–concentration steady state; – not stated.

9NC i.m. at a dose of 4 mg $kg^{-1}$ twice a week (Pantazis et al, 1993*a*). No tumour regrowth nor toxicity occurred during prolonged 9NC administration.

Cell cultures of non-tumorigenic breast cancer cells (MDA-MB-134) and tumorigenic cells (MDA-MB-231) were exposed to 9NC. The non-tumorigenic cells accumulated in $G_2$/M without significant changes in S-fraction. Removal of 9NC from the cultures of non-tumorigenic cells after 120 h resulted in regrowth at a rate similar to untreated cells. In tumorigenic cells exposed to 9NC, there was a marked increase in cells containing a reduced DNA content and going into apoptosis. Removal of 9NC from the cultures of tumorigenic cells after 120 h did not result in regrowth after 120 h (Pantazis et al, 1993*a*).

Experiments with 9NC and 9AC at an i.m. dose of 4 mg $kg^{-1}$ twice weekly in various human breast cancer xenografts resulted in complete tumour regression but, regardless of 9NC continuation or discontinuation, tumorigenic MDA-MB-231 tumours regrew after a period of 50 days of complete tumour regression (Pantazis et al, 1993*b*). This indicates that drug resistance occurs.

Protracted i.v. administration of 9AC to mice innoculated with CLO human breast cancer cells was studied in various schedules. 9AC i.v. daily × 3 every 21 days at dose levels of 0.75 and 1 mg $kg^{-1}$ $day^{-1}$ resulted in tumour regression but, ultimately, with regrowth. This i.v. schedule had no inhibitory effect on tumour progression, unlike the i.m. 9AC 1 mg $kg^{-1}$ administration described earlier. A 5-day period of continuous 9AC administration followed by 2 days' rest was highly effective in tumour inhibition and regression even at a dose of 0.5 mg $kg^{-1}$ $day^{-1}$. 9AC doses of 1 mg $kg^{-1}$ $day^{-1}$ or above were toxic for the animals. Intragastric administration of 9NC and 9AC was studied at different doses and schedules in mice with CLO xenografts. The optimal 9NC and 9AC dose and schedule was 1 mg $kg^{-1}$ $day^{-1}$ for 5 days followed by 2 days' rest. The authors conclude that, for practical reasons, oral administration is the route of choice for 9NC (Pantazis et al, 1993*b*).

Intramuscular administration of 9NC 4 mg $kg^{-1}$ twice a week was efficacious in nude mice bearing human 2774 ovarian cancer (Pantazis et al, 1993*c*). Prolonged exposure of tumorigenic (2774) and non-tumorigenic (DUN) ovarian cancer cells in vitro to a concentration of 1 ng $ml^{-1}$ of 9NC resulted in accumulation of non-tumorigenic cells in $G_2$/M and accumulation of tumorigenic cells containing reduced DNA content and going into apoptosis (Giovanella et al, 1994).

In a human melanoma xenograft model intramuscular administration gave the best anti-tumour effects of 9NC, 9AC and CPT. A dose schedule of 2 mg $kg^{-1}$ $day^{-1}$ ×2 with 1 day rest compared with 1 mg $kg^{-1}$ $day^{-1}$ ×5 with 2 days' rest was more effacicious for CPT and equally effective for 9NC (Pantazis et al, 1994*b*).

Intragastric application of 9AC on a 5-day/week schedule for 3–6 weeks induced complete remission in subcutaneous human xenografts of malignant melanoma and non-small-cell lung carcinoma, and its efficacy was better than that of 20-S-camptothecin (Rubin et al, 1994).

Two observations can be made on these preclinical studies: lower 9NC or 9AC concentrations applied for long periods of treatment are more effective in inducing apoptosis than higher concentrations for short periods. When 9NC initiates the process of apoptosis in tumorigenic cells, this is not reversible, even after removal of the drug. Non-tumorigenic cells are reversibly inhibited as long as drug exposure continues.

Route of administration and dose scheduling of 9NC and 9AC seem to be crucial for optimal anti-tumour responses. Prolonged or intermittent administration of a lower dose of these drugs is most efficacious.

### Clinical studies with prolonged or continuous exposure

### *72-h infusion*

Phase I studies of 9-amino-camptothecin in adult patients with solid tumours have been performed initially with continuous i.v. infusion over 72 h (Table 1). Leucopenia appeared to be the dose-limiting toxicity, together with modest thrombocytopenia. Nausea and vomiting, alopecia, stomatitis and diarrhoea were less frequently reported (Rubin et al, 1994; Takimoto et al, 1994; Dahut et al, 1996). Steady-state plasma concentrations increased linearly with the dose and ranged from 0.9 to 10.6 nm and correlated well with percentage decrease of granulocyte count (Takimoto et al, 1994). In a similar phase I study in children, side-effects were similar, but the MTD in children exceeded that in adults (Langevin et al, 1996).

*© Cancer Research Campaign 1997*

*Prolonged exposure*

Phase I studies with longer infusion durations of 9-AC in adults are ongoing. A continuous i.v. infusion for 120 h weekly for 3 out of every 4 weeks is feasible, with DLT not yet being reached at the dose level of 20 μg $m^{-2}$ $h^{-1}$. The resulting dose intensity is already higher than the dose intensity of the recommended phase II dose of 35 μg $m^{-2}$ $h^{-1}$ over 72 h when given every 2 weeks (Takimoto et al, 1996).

The same holds for continuous infusion of 9AC for 21 consecutive days every 28 days (Hochster et al, 1996*a*). The latter phase I studies suggest that with prolonged infusion a higher dose intensity of 9AC can be achieved. A phase I study with oral 9-NC given for 5 consecutive days every week revealed haematological toxicity as being dose limiting. Non-haematological toxicity was substantial with nausea/vomiting, diarrhoea and haemorrhagic cystitis. An interesting level of anti-tumour activity was reported (Verschraegen et al, 1996).

Further studies on prolonged dosing of oral 9NC and i.v. 9AC are presently on-going.

In summary, dose intensities are higher for 9AC when administered with longer infusion duration. Oral administration of 9NC for 5 consecutive days gives substantial non-haematological toxicity.

### TOPOTECAN

Topotecan (TPT, 9-dimethylaminomethyl-10-hydroxycamptothecin) is a water-soluble potent camptothecin analogue with activity against various tumour types in in vitro and in vivo studies.

### Preclinical studies

*In vitro studies*

In vitro effects of topotecan against cells from biopsy specimens of colorectal, breast, lung, ovarian, renal, gastric cancer and cancers of unknown primary origin were studied with 1-h and with continuous exposure in a human tumour clonogenic assay. With 1-h TPT exposure in vitro, responses were seen in 10% and 25% of assessable tumour specimens at TPT concentrations of 1.0 and 10.0 μg $ml^{-1}$ respectively. Response rates were 34% and 76% at concentrations of 0.1 and 1.0 μg $ml^{-1}$ TPT with continuous exposure (Burris et al, 1992), suggesting that TPT was more active with long-term incubation. Continuous exposure of TPT in vitro showed an initial decrease of the active lactone form of TPT, followed by a stable ratio up to 72 h, which corresponded to 19% of the initial value. The fraction of the lactone form during 1-h exposure is not known, but nevertheless it is very likely that the concentration–time product (dose intensity) is greater for continuous exposure than for 1-h exposure (Burris et al, 1992). This implies that the time period of exposure to topotecan is an even greater determinant of cytotoxicity than anticipated.

*In vivo studies*

Different TPT schedules were studied in female CBA/CaJ immune-deprived mice engrafted with seven colon carcinoma cell lines, six juvenile rhabdomyosarcomas and three osteosarcoma cell lines (Houghton et al, 1991). Initially, TPT was administered intraperitoneally (i.p.) using a schedule of four doses of TPT every 4 days (q4d × 4 schedule). The maximum-tolerated dose (MTD) with this schedule was 12.5 mg $kg^{-1}$ per administration, and TPT caused significant regression in four of five rhabdomyosarcoma xenografts. Subsequently, the effect of TPT was studied as a daily ×5 dose given for 3 consecutive weeks by oral gavage (2 mg $kg^{-1}$ per administration) or daily × 5 for 3 weeks intraperitoneally. Intraperitoneal administration was at least as efficacious as oral dosing but more toxic (Houghton et al, 1991). Intraperitoneal TPT 2 mg $kg^{-1}$ per dose was lethal in more than 15% of the mice, the MTD with intraperitoneal administration was 1.5–1.75 mg $kg^{-1}$ per dose. The effect of prolonged topotecan administration was studied in two moderately responsive xenografts, Rh 12 rhabdomyosarcoma and $VRC_5$ colon adenocarcinoma. Mice bearing Rh 12 rhabdomyosarcoma xenografts were treated with TPT 2.0 or 1.75 mg $kg^{-1}$ per dose $day^{-1}$ × 5 for three courses or a lower dose (1.25 mg $kg^{-1}$ per dose) for up to 20 courses. The prolonged low-dose regimen resulted in complete remission of all tumours without regrowth. The same effect was seen at an even lower dose level of 1.0 mg $kg^{-1}$ per dose, also without significant toxicity. Mice with $VCR_5$ colon adenocarcinoma showed significant tumour reduction with prolonged oral administration of TPT at a dose of 1.0 mg $kg^{-1}$ per dose × 5 for 20 cycles. However, regrowth occurred after 16 weeks.

Additional studies with prolonged exposure schedules in mice bearing xenografts of colon adenocarcinoma, rhabdomyosarcoma and brain tumours showed less toxicity and better anti-tumour activity than dose-intensive short-exposure schedules (Houghton et al, 1995). These in vivo studies show that oral administration is as efficacious as parental application, although the AUC is lower with oral administration.

Furthermore, prolonged intraperitoneal and oral (p.o.) TPT administration resulted in responses of xenografts not responsive to a short-term parental intermittent high-dose schedule (Houghton et al, 1991, 1995).

From these preclinical data, prolonged exposure to topotecan seems a treatment schedule with a potentially higher benefit with regard to anti-tumour activity.

### Clinical studies with prolonged or continuous exposure

*Daily × 5 i.v. administration*

Phase I studies with single i.v. bolus daily for 5 days repeated every 3–4 weeks show a maximum-tolerated dose of 1.5–2.5 mg $m^{-2}$ $day^{-1}$. The dose-limiting toxicity was myelosuppression, in particular neutropenia (Rowinsky et al, 1992; Saltz et al, 1992; Verweij et al, 1993).

Non-haematological toxicities were usually mild and reversible and consisted of nausea, vomiting, fatigue, alopecia and sometimes diarrhoea.

Phase II studies with this daily ×5 TPT regimen every 21 days showed promising response rates in patients with small-cell lung cancer (10–39%) and in pretreated patients with ovarian cancer with response rates ranging from 9.5% to 25% (Kudelka et al, 1993; Schiller et al, 1994; Armstrong et al, 1995; Wanders et al, 1995; Carmichael et al, 1996; Malmstrom et al, 1996; Creemers et al 1997). Other solid tumours, such as melanoma, colon carcinoma, head and neck cancer, renal cell carcinoma, cervix and prostate carcinoma, appear to be much less sensitive to this regimen (Ilson et al, 1993; Roethke et al, 1993; Lynch et al, 1994; Robert et al, 1994; Sugarman et al, 1994; Kraut et al, 1995; Puc et al, 1995; Noda et al, 1996; Perez Soter et al, 1996; Smith et al, 1996). In these phase II studies, CTC grade III–IV neutropenia (32–81%) was reported as being the major toxicity. Thrombocytopenia CTC grade III–IV is infrequent. Anaemia greater than CTC grade II was reported in 27–60%.

© Cancer Research Campaign 1997

**Table 2** Topoisomerase I inhibitors – continuous/prolonged administration in solid tumours (topotecan)

| Drug | Dose (mg m$^{-2}$) schedule | Number of patients | Cp-ss | MTD | DLT | Reference |
|---|---|---|---|---|---|---|
| Topotecan i.v. | 2.5–5.0<br>24 h q 3 wk | 15 | – | 4 mg m$^{-2}$ 24 h$^{-1a}$<br><br>5 mg m$^{-2}$ 24 h$^{-1b}$ | Neutropenia<br>Thrombopenia | Recondo (1991) |
| Topotecan i.v. | 2.5–5.0<br>24 h q 3 wk | 10 | 4–10 ng ml$^{-1}$ | – | Neutropenia | Reid (1992) |
| Topotecan i.v. | 2.5–10.5<br>24 h q 3 wk | 22 | 20 ng ml$^{-1}$ | 8.4 mg m$^{-2}$ 24 h$^{-1}$ | Neutropenia | ten Bokkel Huinink (1992) |
| Topotecan i.p. | 3–4<br>24 h q 4 wk | 12 | – | 4 mg m$^{-2}$ 24 h$^{-1}$ | Neutropenia | Plaxet et al (1993) |
| Topotecan i.v. | –/72 h q 1 wk<br>–/72 h q 2 wk | 12<br>7 | –<br>– | 2 mg m$^{-2}$ 72 h$^{-1}$<br>2.6 mg m$^{-2}$ 72 h$^{-1}$<br>72 h | Neutropenia<br>Neutropenia | Sabiers et al (1993) |
| Topotecan i.v. + G-CSF | 10–15<br>24 h q 3 wk | 13 | – | 4 mg m$^{-2a}$<br>10 mg m$^{-2b}$ | Neutropenia<br>(+G-CSF: Thrombopenia) | Abbruzzese (1993) |
| Topotecan i.v. | 2.0–7.5<br>24 h q 3 wk | 29 | 18.2 ± 3.7 nM | 7.5 mg m$^{-2}$ | Neutropenia<br>Thrombopenia | Blaney (1993) |
| Topotecan i.v. | 1.0–2.0<br>24 h q 1 wk | 32 | 4.7–11.4 nM | 1.75 mg m$^{-2}$ 24 h$^{-1}$ | Neutropenia | Haas (1994) |
| Topotecan i.v. | 0.75–1.9 day$^{-1}$<br><br>72 h q 3 wk | 27 | 3.1 ± 1.4 ng ml$^{-1}$<br>1.0 mg m$^{-2}$ d$^{-1}$ | 1.3 mg m$^{-2}$ | Neutroperia | Pratt (1994) |
| Topotecan i.v. | 0.17–0.68 day$^{-1}$<br>120 h q 3 wk<br>0.68–1.6 day$^{-1}$<br>72 h q 3 wk | 14<br><br>32 | 5.5 ng ml$^{-1}$<br><br>2.0 ng ml$^{-1}$ | 0.68 mg m$^{-2}$ day$^{-1}$<br><br>1.6 mg m$^{-2}$ day$^{-1}$ | Thrombopenia<br><br>Neutropenia | Burris (1994) |
| Topotecan i.v. | 0.2–0.7<br>21 d q 28 d | 44 | – | 0.53 mg m$^{-2}$ day$^{-1}$ | Thrombopenia + Neutropenia | Hochster et al (1994) |
| Topotecan i.v. | 0.6 day$^{-1}$ 21 d | 9 | – | – | Neutropenia<br>Thrombopenia | Khater (1995) |
| Topotecan i.v. | 0.4 day$^{-1}$<br>21 d q 28 d | 16 | – | –<br>(Phase II) | – | Hochster et al (1996) |
| Topotecan i.v. | 0.8–1.1 day$^{-1}$<br>21 d q 28 d | 12<br>Pediatric | – | 0.8 mg m$^{-2}$ day$^{-1}$ | Thrombopenia | Bowman et al (1996) |

[a]Chemotherapy pretreated. [b]Non-pretreated. i.v., intravenous; i.p., intraperitoneal, p.o. oral; MTD, maximum-tolerated dose; DLT, dose-limiting toxicity; CP-ss, plasma–concentration steady state; – not stated.

### *Prolonged exposure*

Continuous infusion of topotecan has been studied in various schedules: a 24 h infusion weekly and every 3 weeks; a 72 h infusion administered weekly, every 14 days and every 21 days; a 120-h infusion every 3–4 weeks; and a 21-day continuous infusion administered every 28 days (Table 2).

In one study, TPT was administered intraperitoneally for 24 h every 4 weeks (Plaxe et al, 1993). Studies with continuous infusion of topotecan of 72 h or more show mild non-haematological toxicities (nausea, vomiting, alopecia). Dose-limiting toxicity is always leucocytopenia, more often with associated thrombocytopenia than with the daily × 5 i.v. bolus. Anaemia requiring blood transfusions and thrombocytopenia with platelet transfusions are particular problems related to these schedules. In phase II studies in paediatric patients and adults with acute leukaemia, continuous infusion of TPT for 120 h resulted in severe mucositis as DLT (Kantarjian et al, 1993; Furman et al, 1996).

In a phase I study with continuous intravenous topotecan administration for 21 days every 28 days in 44 patients with solid tumours, the MTD was 0.53 mg m$^{-2}$ day$^{-1}$, with myelosuppression as DLT (Hochster et al, 1994). The steady-state lactone TPT concentration was low, approximately 4 ng ml$^{-1}$. No consistent relationship was found between drug level and haematological toxicity. Partial tumor responses were noted in two patients with ovarian cancer, one patient with breast cancer, one patient with renal cell cancer and one patient with non-small-cell lung cancer (NSCLC) (Hochster et al, 1994). Blood transfusions and platelet transfusions were necessary in 45% and 11% of patients respectively. The authors concluded that a 21-day infusion of TPT is generally well tolerated with minimal non-haematological toxicity. In a phase II study with this regimen in patients with progressive ovarian cancer after platinum-containing chemotherapy, response rate was 37% and neutropenia was the major toxicity (31%). Blood transfusions needed to be given to 50% of patients (Hochster et al, 1996*b*). Further phase II studies with the 21-day continuous infusion of TPT are ongoing.

The bioavailability of oral TPT varies from 32% to 44% with relatively limited intrapatient variation (Kuhn et al, 1995; Schellens et al, 1996). Oral TPT was studied in paediatric patients with solid tumours in a phase I study with two different dose schedules. In one dose schedule, TPT was administered orally every day for 21 days out of every 28 days; in the second schedule, oral TPT was given 5 days on and 2 days off for 15 total doses. In the 21-day schedule oral bioavailability was 46 ± 22% at 0.8 mg m$^{-2}$ and 34 ± 14% at dose level 1.1 mg m$^{-2}$. DLT of both schedules is thrombocytopenia, and myelosuppression is well correlated with systemic exposure to oral TPT (Bowman et al, 1996). Thus, in vitro studies show that time period of exposure to

© Cancer Research Campaign 1997

**Table 3** Topoisomerase I inhibitors – continuous/prolonged administration in solid tumours (CPT-11)

| Drug | Dose (mg m²) Schedule | Number of patients | Cp-ss | MTD | DLT | Reference Year |
|---|---|---|---|---|---|---|
| CPT-11 i.v. | 125–225 every other wk | 20 | – | ≥ 200 mg m$^{-2}$ | Not yet reached | Rothenberg (1996) |
| CPT-11 i.v. bolus. | 33–115 day$^{-1}$ 3 d q 3 wk | 46 | 2034 ng ml$^{-1}$ | 115 mg m$^{-2}$ | Neutropenia + diarrhoea | Catimel et al (1995) |
| CPT-11 i.v. | 5–40 day$^{-1}$ 120 h q 3 wk | 36 | 6.8–10.5 ng ml$^{-1}$ (SN 38) | 40 mg m$^{-2}$ day$^{-1}$ | Neutropenia diarrhoea | Ohe et al (1992) |

i.v., Intravenous; i.p., intraperitoneal; p.o, oral; MTD, maximum-tolerated dose; DLT, dose-limiting toxicity; Cp-ss, plasma–concentration steady state; –, not stated.

topotecan is an important determinant of cytotoxicity. In vivo studies with human xenografts with prolonged administration of topotecan show better anti-tumour activity. In patients with solid tumours, continuous infusion of TPT is well tolerated, and tumour responses are being reported. Phase I studies with an oral formulation of TPT in adult patients with solid tumours are ongoing.

### IRINOTECAN (CPT-11)

CPT-11 (7-ethyl-10 [4-(piperidino)-1-piperidino]carboxyloxy-camptothecin) is a water-soluble analogue of camptothecin. CPT-11 has little inherent anti-tumour activity in vitro, but it is converted to SN-38, a metabolite that is 1000-fold more potent than the parent compound in vitro (Kawato et al, 1991; Kanzawa et al, 1993).

### Preclinical studies

#### *In vivo studies*

CPT-11 has been studied in human tumour xenografts with chemorefractory colon-carcinoma, chemoresponsive rhabdomyosarcoma and sublines of rhabdomyosarcoma with in vivo resistance to vincristine, melphalan and topotecan, as well as with three paediatric brain tumours (Houghton et al, 1993, 1995). As a single i.v. administration at the maximum-tolerated dose (50 mg kg$^{-1}$), CPT-11 had no inhibitory effect on any colon carcinoma xenograft; however, when administered for one cycle i.v. at a dose of 10–40 mg kg$^{-1}$ per dose daily ×5 for 2 consecutive weeks, it demonstrated significant activity against five of eight colon carcinoma models. Rhabdomyosarcomas and two xenografts (Rh 18 rhabdomyosarcoma and $VRC_5$ colon adenocarcinoma), resistant in vivo to topotecan, were also highly responsive to this schedule (Houghton et al, 1993). To determine whether prolonged periods of treatment were more effective CPT-11 was administered as before as a daily ×5 schedule for 2 weeks, but the cycles were repeated every 21 days for a total of three cycles. The MTD was 10 mg kg$^{-1}$ day$^{-1}$. Complete regression of all $VRC_5$ colon tumours was achieved at 5–10 mg kg$^{-1}$ per dose. CPT-11, given as a protracted schedule at 5 mg kg$^{-1}$ day$^{-1}$, showed greater activity than a shorter intense therapy at 40 mg kg$^{-1}$ per dose.

A single cycle of CPT-11 was only modestly active at a dose of 40 mg kg$^{-1}$ in 4 of 25 Rh 12 rhabdomyosarcoma xenografts whereas three cycles of therapy at 10 mg kg$^{-1}$ day$^{-1}$, daily × 5, resulted in complete regression in 12 of 13 tumours. Similar results were obtained in colon carcinoma and human brain tumour xenografts (Houghton et al, 1993).

Thus, protracted therapy with low-dose CPT-11 has increased therapeutic efficacy compared with more toxic short-term schedules.

### Clinical studies with prolonged or continuous administration

In a phase I study with CPT-11 given as a 5-day continuous infusion every 3 weeks, the dose was escalated from 5 to 40 mg m$^{-2}$ day$^{-1}$ (Ohe et al, 1992) (Table 3). Dose-limiting toxicity consisted of CTC grade III–IV diarrhoea. Toxic effects greater than CTC grade II included diarrhoea (69%), nausea and vomiting (58%), leucopenia (25%), anaemia (25%), thrombocytopenia (6%) and hepatic dysfunction (14%). Diarrhoea was dose dependent, in contrast to the white blood cell nadir, which was not dose dependent (Ohe et al, 1992). In another phase I study, CPT-11 was administered intravenously over 30 min for 3 consecutive days every 3 weeks. Both leucopenia and diarrhoea were dose limiting at a dose of 115 mg m$^{-2}$ day$^{-1}$ (Catimel et al, 1995).

In limited studies with low-dose schedules of CPT-11 once daily × 3, once daily × 5 and twice daily × 7, anti-tumour responses were reported in patients with leukaemia and lymphomas (Ohno et al, 1990; Tsuda et al, 1992).

From small studies in ovarian and cervical cancer, it was suggested that there were no significant differences between schedules concerning efficacy, but clearly these data need further confirmation (Takeuchi et al, 1991*a* and *b*). Response rates in patients with NSCLC treated with CPT-11 at a dose of 200 mg m$^{-2}$ every 3–4 weeks or 100 mg m$^{-2}$ weekly do not seem to differ (Nakai et al, 1991; Negoro et al, 1991). In patients with solid tumours, the dose schedule apparently does not seem to be crucial in efficacy of the drug. However, CPT-11 may have more efficacy when administered at lower doses for a longer time to patients with malignant lymphoma.

An oral formulation of CPT-11 has been tested on a daily × 5 schedule every 3 weeks with diarrhoea and neutropenia as dose-limiting toxicities (Drengler et al, 1996).

### GI147211

GI147211, (7-(4-methyl piperazinomethylene)10,11-ethylenedioxy-20-(S)-camptothecin) is a water-soluble analogue of camptothecin. The water-solubilizing groups were introduced on position 7 in the B ring.

### Preclinical studies

GI147211 appeared to have anti-tumour activity in vitro as well as in vivo (Emerson et al, 1995). In these studies, the dose schedule of twice a week administration for 5 weeks did not appear optimal. Recent data demonstrate that GI147211 is more active

© Cancer Research Campaign 1997

**Table 4** Topoisomerase I inhibitors – continuous/prolonged administration in solid tumours (GI147211)

| Drug | Dose (mg m$^{-2}$) Schedule | Number of of patients | Cp-ss | MTD | DLT | Reference |
|---|---|---|---|---|---|---|
| GI147211 i.v. | 0.3–0.5 day$^{-1}$ 7–21 d q 28 d | 38 | 0.1–0.35 ng ml$^{-1}$ | 0.5 mg m$^{-2}$ d$^{-1}$ × 21 | Neutropenia Thrombopenia | Khater et al (1996) |
| GI147211 i.v. | 72 h q 28 d? | 36 | – | 1.5 mg m$^{-2}$ d$^{-1a}$ 2.0 mg m$^{-2}$ d$^{-1b}$ | Myelosuppression | O'Dwyer et al (1995) |

[a]Chemotherapy pretreated. [b]Non-pretreated. i.v., Intravenous; i.p., intraperitoneal, p.o, oral; MTD, maximum-tolerated dose; DLT, dose-limiting toxicity; Cp-ss, plasma–concentration steady state; –, not stated.

when administered at higher doses using an every 4 days schedule for a total of three doses (Emerson et al, 1994). Again, dose scheduling seems to be important for an optimal anti-tumour effect.

### Clinical studies with prolonged or continuous administration

### *Daily ×5 i.v. administration*

Three phase I studies with intravenous GI14721 have been performed, two studies with a 30-min GI147211 infusion once daily for 5 consecutive days every 3 weeks, a third study with GI147211 given as a 72-h continuous infusion (Eckardt et al, 1995; O'Dwyer et al, 1995; Gerrits et al, 1996). In all studies, AUC increased with dose in a linear fashion, and dose-limiting toxicity consisted of leucocytopenia as well as thrombocytopenia. Non-haematological toxicity was mild and there was no diarrhoea or haemorrhagic cystitis. Preliminary results of phase II studies show anti-tumour activity in ovarian cancer and small-cell lung cancer (Wanders et al, 1996).

### *Prolonged exposure*

A phase I study with continuous infusion of GI147211 has been performed with doses ranging from 0.3 to 0.5 mg m$^{-2}$ day$^{-1}$ for 7, 14 and 21 days. DLT reached at 0.5 mg m$^{-2}$ day$^{-1}$ consisted of neutropenia and thrombocytopenia. Non-haematological toxicities CTC grade ≥ II consisted of nausea, vomiting, dyspepsia, fatigue and diarrhoea. Pharmacokinetics of GI147211 showed mean steady-state concentrations ranging from 0.1 to 0.35 ng ml$^{-1}$. The total body clearance was similar to the clearance with shorter infusions (Khater et al, 1996) (Table 4).

### DISCUSSION AND CONCLUSION

Topoisomerase I inhibitors are a class of drugs with a broad anti-tumour activity, even against previously chemotherapy-resistant tumours. The issues concerning drugs scheduling are many, and one of the conclusions from this review could be that there is no true consistency in the use of schedules and models in preclinical studies. It would be worthwhile to try to achieve this consistency in the development of drugs such as these. Clearly, many of the relevant questions on scheduling can already be answered in in vitro studies, such as the ones that have been performed with topotecan. With appropriate in vitro studies, one could easily mimic potential clinical application schedules. Following in vitro studies, in vivo studies could be performed taking the data from the in vitro studies into account. Obviously, long-term infusional application in animal models is difficult to achieve but, on the other hand, many of the performed in vivo studies, because of their diversity, do not result in conclusive evidence. With a consistent approach in preclinical studies, one could also avoid the need to perform too many clinical studies on scheduling. We also recommend performing the clinical phase I and II studies with inclusion of pharmacokinetic/pharmacodynamic (PK/PD) relationship studies. A good example of this can be found in a yet unpublished study relating levels of topoisomerase I inhibitors to parameters such as decreased cleavable complex formation. Making use of the appropriate combinations of clinical studies with PK/PD studies, the number of studies necessary could easily be reduced. Also, such studies would answer the question of whether thresholds exist for the effect of topoisomerase I inhibitors in conjunction with exposure duration. The preliminary results from the above reviewed phase I and phase II studies indicate that prolonged administration with topoisomerase I inhibitors is feasible in patients with cancer. However, unfortunately, the optimal dose and schedule of the various agents available remain to be elucidated. Although preliminary results are encouraging and warrant further clinical exploration, the concept should still be considered as being investigational.

### REFERENCES

Abbrazzese JL, Madden T, Schmidt S, Eaton G and Raber MN (1993) Phase I trial of topotecan (TT) administered by 24-hour infusion without and with G-CSF (abstract 1957). Proc Am Assoc *Cancer Res* **34**: 329

Aller P, Rius C, Mata F, Zorilla A, Carbanas C, Bellon T and Bernabeu C (1992) Camptothecin induces differentiation and stimulates the expression of differentiation-related genes in U-937 human promonocytic leukemia cells. *Cancer Res* **52**: 1245–1251

Andoh T, Ischii K, Suzuki Y, Ikegami Y, Kusunoki Y, Takemoto Y and Okada K (1987) Characterization of a mammalian mutant with a camptothecin-resistant DNA topoisomerase I. *Proc Natl Acad Sci USA* **84**: 5565–5569

Aogi K, Nishiyama M, Hirabayashi N, Toge T, Okada K, Kusano T and Ando T (1994) Establishment of a new multidrug-resistant cell line induced by continuous exposure to CPT-11. *Proc Am Assoc Cancer Res* **35**: 451

Armstrong D, Rowinsky E, Donehower R, Rosenshein N, Walczak J and McGuire W (1995) A phase II trial of topotecan as salvage therapy in epithelial ovarian cancer (Abstract 769). *Proc Am Soc Clin Oncol* **14**: 275

Blaney SM, Balis FM, Cole DE, Craig C, Reid JM, Ames MM, Krailo M, Reaman G, Hammond D and Poplack DG (1993). Pediatric phase I trial and pharmacokinetic study of Topotecan administered as a 24-hour continuous infusion. *Cancer Res* **53**: 1032–1036

ten Bokkel Huinink WW, Rodenhuis S, Beijnen J, Dubbelman R and Koier I (1992). Phase I study of the topoisomerase I inhibitor topotecan (SK & F 104864-A) (abstract 260). *Proc Am Soc Clin Oncol* **11**: 110

Burris HA, Awada A, Kuhn JG, Eckardt JR, Cobb PW, Rinaldi DA, Fields S, Smith L and Von Hoff DD (1994) Phase I and pharmacokinetic studies of topotecan administered as a 72 or 120 h continuous infusion. *Anti-Cancer Drugs* **5**: 394–402

© Cancer Research Campaign 1997

D'Arpa P, Beardmore C and Liu LF (1980) Involvement of nucleic acid synthesis in cell killing mechanisms of topoisomerase poisons. *Cancer Res* **50**: 6919–6924

D'Arpa P and Liu LF (1989) Topoisomerase-targeting antitumor drugs. *Biochem Biophys Acta* **989**: 163–177

Del Bino G and Darzynkiewicz Z (1991) Camptothecin, teniposide, or 4'- (9-acridinylamino)-3-methanesulfon-m-anisidide, but not mitoxantrone or doxorubicine induces degradation of nuclear DNA in the S phase of HI-60 cells. *Cancer Res* **51**: 1165–1169

Bowman LC, Stewart CF, Zamboni WC, Crom WR, Luo X, Heideman R, Houghton PJ, Meyer WH and Pratt CB (1996) Toxicity and pharmacodynamics of oral topotecan in pediatric patients with relapsed solid tumors. *Proc Am Soc Clin Oncol* **15**: 462

Bruno S, Giaretti W and Darzynkiewicz Z (1992) Effect of camptothecin on mitogenic stimulation of human lymphocytes: involvement of DNA topoisomerase I in cell transition from $G_0$ to $G_1$ phase of the cell cycle and in DNA replication. *J Cell Physiol* **151**: 478–486

Burris HA, Hanauske AR, Johnson RK, Mashall MH, Kuhn JG, Hilsenbeck SG and van Hoff DD (1992) Activity of topotecan, a new topoisomerase I inhibitor against human tumor colony forming units in vitro. *J Natl Cancer Inst* **23**: 1816–1820

Carmichael J, Gordon A, Malfetano J, Gore M, Spaczynski M, Davidson N, Savage J, Clarke Pearson D, Hudson I, Broom C and Ten Bokkel Huinink W (1996) Topotecan, a new active drug vs paclitaxel in advanced epithelial ovarian carcinoma: international topotecan study group trial. *Proc Am Soc Clin Oncol* **15**: 283

Catimel G, Chabot GG, Guastalla JP, Dumortier A, Cote C, Engel C, Gouyette A, Mathieu-Boue A, Mahjoubi M and Clavel M (1995) Phase I and pharmacokinetic study of irinotecan (CPT-11) administered daily for three consecutive days every three weeks in patients with advanced solid tumors. *Ann Oncol* **6**: 133–140

Champoux J (1976) Evidence for an intermediate with a single-strand break in the reaction catalyzed by the DNA untwisting enzyme. *Proc Natl Acad Sci USA* **73**: 3488–3491

Chen AY, Yu C, Potmesil M, Wall ME, Wani MC and Liu LF (1991) Camptothecin overcomes MDR-1 mediated resistance in human carcinoma cells. *Cancer Res* **51**: 6039–6044

Chou S, Kaneko M, Nakaya K and Nakamura Y (1990) Induction of differentiation of human and mouse myeloid leukemia cells by camptothecin. *Biochem Biophys Res Commun* **166**: 160–167

Covey JM, Jaxel C, Kohn KW and Pommier Y (1989) Protein linked DNA strand breaks induced in mammalian cells by camptothecin an inhibitor of topoisomerase I. *Cancer Res* **49**: 5016–5022

Creaven PJ, Allen LM and Muggia FM (1972) Plasma camptothecin (NSC 100880) levels during a 5 days course of treatment: relation to dose and toxicity. *Cancer Chemother Rep* **56**: 573–578

Creemers GJ, Bolis G, Gone M, Scarfone G, Lacave AJ, Guastalla JP, Despos R, Favelli G, Kreinberg R, Van Belle S, Hudson I, Verweij J and Ten Bokkel Huinink WW (1996) Topotecan, an active drug in the second-line treatment of epithelial ovarian cancer: results of a large European phase II study. *J Clin Oncol* **14**: 3056–3061

Dahut W, Harold N, Takimoto C, Allegra C, Chen A, Hamilton M, Arbuck S, Sorensen M, Grollman F, Nakashina H, Lieberman R, Liang M, Corse W and Corem J (1996) Phase I and pharmacologic study of 9-aminocamptothecin given by 72-hour infusion in adult cancer patients. *J Clin Oncol* **14**: 1236–1244

Daoud SS, Fetouh MI and Giovanella BC (1995) Antitumor effect of liposome-incorporated camptothecin in human malignant xenografts. *Anticancer Drugs* **6**: 83–93

Drengler R, Burris H, Dietz A, Eckhardt J, Eckhardt G, Hodges S, Kraynak M, Kuhn J, Peacock N, Rinaldi D, Rizzo J, Rodriguez G, Schaaf L, Smith L, Thurman A and Von Hoff D (1996) A phase I trial to evaluate orally administered Irinotecan HCl (CPT-11) given daily × 5 every 3 weeks in patients with refractory malignancies. *Proc Am Soc Clin Oncol* **15**: 489

Drewinko B, Freireich EJ and Gottlieb JA (1974) Lethal activity of camptothecin sodium on human lymphoma cells. *Cancer Res* **34**: 747–750

Eckardt JR, Rodriguez GI, Burris HA, Wissel PS, Fields SM, Rothenberg ML, Smith L, Thurman A, Kunka RL, De Pee SP, Littlefield D, White LJ and Von Hoff DD (1995) A Phase I and pharmacokinetic study of the topoisomerase I inhibitor GG211. Abstract 1544. *Proc Am Soc Clin Oncol* **14**: 476

O'Dwyer P, Cassidy J, Kunka R, Pagarez L, Kaye S, De Pee S, Littlefield D, De Maria D, Selinger K, Beranke P, Collis P and Wissel P (1995) Phase I trial of GG211, a new topoisomerase inhibitor using a 72 hour continuous infusion (CI). *Proc Am Soc Clin Oncol* **14**: 471

Emerson DL, Mcintyre G, Luzzio M and Wissel PS (1994) Preclinical antitumor activity of a novel water-soluble camptothecin analog (GI147122 c). *Ann Oncol* **5** (suppl. 5): 185

Emerson DL, Besterman JM, Braun R, Evans MG, Leitner PP, Luzzio MJ, Shaffer JE, Sternbach DD, Uehling D and Vuong A (1995) In vivo antitumor activity of two new seven substituted water-soluble camptothecin analogues. *Cancer Res* **55**: 603–609

Eng WK, Faucette L, Johnson RK and Stenglanz R (1989) Evidence that DNA topoisomerase is necessary for the cytotoxic effects of campotothecin. *Mol Pharmacol* **34**: 755–760

Furman WL, Balur SD, Pratt CB, Rivera GK, Evans WE and Stewart CF (1996) Escalating systemic exposure of continuous infusion Topotecan in children with recurrent acute leukemia. *J Clin Oncol* **14**: 1404–1511

Gerrits CJH, Creemers GJ, Schellens JHM, Wissel PS, Planting AST, Kunka R, Selinger K, De Boer-Dennert M, Marijnen Y, Harteveld M and Verweij J (1996) Phase I and pharmacological study of the new topoisomerase I inhibitor GI147211, using a daily ×5 intravenous administration. *Br J Cancer* **73**: 744–750

Giovanella BC, Stehlin JS, Wall ME, Wani MC, Nicholas AW, Liu LF, Silber R and Potmesil M (1989) DNA-topoisomerase I-targeted chemotherapy of human colon cancer in Xenografts. *Science* **246**: 1046–1048

Giovanella BC, Hinz HR, Kozielski AJ, Stehlin JS, Silber R and Potmesil M (1991) Complete growth inhibition of human cancer xenografts in nude mice by treatment with 20-(S)-camptothecin. *Cancer Res* **51**: 3052–3055

Giovanella BC, Stehlin JS, Hinz HR, Vardeman D, Mendoza JT and Potmesil M (1994) Studies of time/dose intensity in treatment of human cancer xenografts with camptothecin analogues (abstract 2713). *Proc Am Assoc Cancer Res* **35**: 455.

Gottlieb JA and Luce JK (1972) Treatment of malignant melanoma with camptothecin (NSC 100880). *Cancer Chemother Rep* **56**: 103–105

Gottlieb JA, Guarino AM, Call JB, Oliverio VT and Block JB (1970) Preliminary pharmacologic and clinical evaluation of camptothecin sodium (NSC 100880). *Cancer Chemother Rep* **54**: 461–470

Guarino AM, Anderson JB and Starkweather DK (1973) Pharmacologic studies of camptothecin (NSC-100880): distribution, plasma protein binding and biliary excretion. *Cancer Chemother Rep* **57**: 125–140

Gupta RS, Gupta R, Eng B, Lock RB, Ross WE, Hertzberg RP, Caranfa MJ and Johnson RK (1988) Camptothecin-resistant mutants of Chinese hamster ovary cells containing a resistant form of topoisomerase I. *Cancer Res* **48**: 6404–6410

Haas NB, LaCreta FP, Walczak J, Hudes GR, Brennan JM, Ozois RF and O'Dwyer PJ (1994) Phase I/Pharmacokinetic study of topotecan by 24 hour continuous infusion weekly. *Cancer Res* **54**: 1220–1226

Hendricks CB, Rowinsky EK, Grochow LD, Donehower RC and Kaufmann SH (1992) Effect of P-glycoprotein expression on the accumulation and cytotoxicity of topotecan (SK&F 104864) a new camptothecin analogue. *Cancer Res* **52**: 2268–2278

Hinz HR, Harris NJ, Natelson EA and Giovanella BC (1994) Pharmacokinetics of the in vivo and in vitro conversion of 9-nitro-20-(S)-camptothecin to 9-amino-20-(S)-camptothecin in humans, dogs and mice. *Cancer Res* **54**: 3096–3100

Hochster H, Liebes L, Speyer J, Sorich J, Taubes B, Oratz R, Wernz J, Chachoua A, Raphael B, Vinci RZ and Blim RH (1994) Phase I trial of low-dose continuous topotecan infusion in patients with cancer: an active and well-tolerated regimen. *J Clin Oncol* **12**: 553–559

Hochster H, Potmesil M, Liebs L, Sorich J, Taubes B, Dewey D, Oratz R, Chachoua A and Speyer J (1996*a*) A phase I study of 9-amino-camptothecin (9AC) by prolonged infusion over 21 days. NCI-EORTC 9th Symposium on New Drugs in Cancer Therapy. p. 130, Amsterdam

Hochster H, Speyer J, Wadler S, Runowicz C, Wallach R, Oratz R, Chacoua A, Sorich J, Taubes B, Ludwig E, Broom C and Blum R (1996*b*) Phase II study of topotecan 21 day infusion in platinum-treated ovarian cancer: a highly active regimen. *Proc Am Soc Clin Oncol* **15**: 285

Horwitz SB (1974) Novel inhibitors of RNA synthesis. *Fed Proc* **33**: 2281–2287

Horwitz SB and Horwitz MS (1973) Effects on Camptothecin on the breakage and repair of DNA during the cell cycle. *Cancer Res* **33**: 2834–2836

Horwitz SB, Chang CK and Grollman AP (1971) Studies on camptothecin I. *Mol Pharmacol* **7**: 632–644

Houghton PJ, Chesire PJ, Myer L, Stewart CF, Synold TW and Houghton JA (1991) Evaluation of 9-dimethylaminomethyl-10-hydroxy-camptothecin (topotecan) against xenografts derived from adult and childhood tumors. *Cancer Chemother Pharmacol* **31**: 229–239

Houghton PJ, Cheshire PJ, Hallman JD, Bissery MC, Mathieu-Boue A and Houghton HA (1993) Therapeutic efficacy of topoisomerase I inhibitor 7-ethyl-10-(4-[1-piperidino-]-1 piperidino)-carbonyloxy-camptothecin against human tumor xenografts: lack of cross-resistance in vivo in tumors with acquired

© Cancer Research Campaign 1997

resistance to the topoisomerase I inhibitor 9- dimethylaminomethyl-10-hydroxy-camptothecin. *Cancer Res* **53**: 2823–2829

Houghton PJ, Chesire PJ, Hallman JD, Lutz L, Friedman HS, Danks MK and Houghton JA (1995) Efficacy of topoisomerase I inhibitor topotecan and irinotecan administered at low dose levels in protracted schedules to mice bearing xenografts of human tumors. *Cancer Chemother Pharmacol* **36**: 393–403

Hsiang YH and Liu LF (1988) Identification of mammalian DNA topoisomerase I as an intracellular target of the anticancer drug camptothecin. *Cancer Res* **48**: 1722–1726

Hsiang YH, Likan MG and Liu LF (1989) Arrest of replication forks by drug stabilized topoisomerase-I-DNA cleavable complexes as a mechanism of cell killing by camptothecin. *Cancer Res* **49**: 5077–5082

Hwang JL, Shyy SH, Chen AY, Juan CC and Whang-Peng J (1989) Studies of topoisomerase specific antitumor drugs in human lymphocytes using rabbit antisera against recombinant human topoisomerase II polypeptide. *Cancer Res* **49**: 958–962

Hwong CL, Chen MS and Hwang J (1989) Phorbol ester transiently increases topoisomerase I mRNA levels in human skin fibroblasts. *J Biol Chem* **264**: 14923–14926

Hwong CL, Chen CY, Shang HF and Hwang J (1993) Increased synthesis and degradation of DNA topoisomerase I during the initial phase of human T-lymphocyte proliferation. *J Biol Chem* **268**: 18982–18986

Ilson D, Motzer RJ, O'Moore P, Nanus D and Bosl GJ (1993) A phase II trial of topotecan in advanced renal cell carcinoma. *Proc Am Soc Clin Oncol* **12**: 248

Jaxel CJ, Kohn KW, Wani MC, Wall ME and Pommier Y (1989) Structure activity study of the action of camptothecin derivatives on mammalian topoisomerase I: evidence for a specific receptor site and a relation to antitumor activity. *Cancer Res* **49**: 1465–1469

Juan C, Hwang J, Liu A, Whang-Peng J, Knutsen T, Huebner K, Croce C, Zhang H, Wang J and Liu L (1988) Human DNA topoisomerase I is encoded by a single copy gene that maps to chromosome region 20q12–13.2. *Proc Natl Acad Sci USA* **85**: 8910–8913

Kantarjian HM, Beran M, Ellis A, Zwelling L, O'Brien S, Cazenave L, Koller C, Rios MB, Plunkelt W, Keatin M and Estey EM (1993) Phase I study of Topotecan, a new topoisomerase I inhibitor in patients with refractory or relapsed acute leukemia. *Blood* **81**: 1145–1151

Kanzawa F, Sugimoto Y, Minato K, Kasahara K, Bungo M, Nakagawa K, Fujiwara Y, Liu LF and Saijo N (1990) Establishment of a camptothecin analogue (CPT-11)-resistant cell line of human non-small cell lung cancer: characterization and mechanism of resistance. *Cancer Res* **50**: 5919–5924

Kanzawa F, Kondoh H, Kwan S and Saijo N (1993) Role of carboxyl esterase on metabolism of camptothecin analog (CPT-11) in non-small cell lung cancer cell line PC-7 cells. *Proc Am Assoc Cancer Res* **33**: 427

Kaufmann SH (1989) Induction of endonucleolytic DNA cleavage in human acute myelogenous leukemia cells by etoposide, camptothecin, and other cytotoxic anticancer drugs. A cautionary note. *Cancer Res* **49**: 5870–5878

Kawato Y, Aonuma M, Hirota Y, Kuga H and Sato K (1991) Intracellular roles of SN-38, a metabolite of the camptothecin derivative CPT-11, in the antitumor effect of CPT-11. *Cancer Res* **51**: 4187–4191

Kessel D (1971) Effects of camptothecin on RNA synthesis in leukemia L 1210 cells. *Biochim Biophys Acta* **246**: 225–232

Kharbanda S, Rubin E, Gunji H, Hinz H, Giovanella B, Pantazis P and Dufe D (1991) Camptothecin and its derivatives induce expression of the c-jun protooncogene in human myeloid leukemia cells. *Cancer Res* **51**: 6636–6642

Khater C, Yao KS, Green F,l Halbherr T, Raskay B, Scher R and O'Dwyer PJ (1995) Interindividual variation in topoisomerase I expression and topotecan toxicity (abstrat 2687). *Proc Am Assoc Cancer Res* **36**: 450

Khater C, Twelves C, Grochow L, De Maria D, Paz-Ares L, Littlefield D, Prittchard JF, Wissel P, Kaye S and O'Dwyer PJ (1996) Phase I trial of the topisomerase I inhibitor GG211 as a 21 day continuous infusion. *Proc Am Soc Clin Oncol* **15**: 483

Kjeldsen E, Bonven BJ, Andoh T, Ischii K, Okada K, Bolund L and Westergaard O (1988) Characterization of a camptothecin-resistant human DNA topoisomerase I. *J Biol Chem* **263**: 3912–3916

Kraut EH, Stanbus A, Mayernich D, King G and Balcerzak SD (1995) Phase II trial of topotecan in metastatic malignant melanoma. *Proc Am Assoc Cancer Res* **36**: 238

Kudelka A, Edwards C, Freedman R, Wallin B, Hord M, Howell E, Harper K, Raber M and Kavanagh J (1993) An open phase II study to evaluate the efficacy and toxicity of topotecan administered intravenously as 5 daily infusions every 21 days to women with advanced epithelial ovarian carcinoma (abstract 821) *Proc Am Soc Clin Oncol* **12**: 259

Kuhn J, Dizzo J, Eckardt J, Fields S, Cobb P, Rodriguez G, Rinadi D, Drendgler R, Smith L, Peacock N, Thurman A, Delacruz P, Hodges S, Von Hoff D and Burris H (1995) Phase I bioavailability study of oral topotecan (abstract 1538). *Proc Am Soc Clin Oncol* **14**: 474

Langevin AM, Casto DT, Kuhn JG, Thomas PJ and Vietti T (1996) A phase I trial of 9-amino-camptothecin in children with refractory solid tumors. A pediatric oncology group study. NCI-EORTC 9th symposium on new drugs in cancer therapy. 130

Ling YH, Tseng MT and Nelson JA (1991) Differentiation induction of human promyelocytic leukemia cells by 10-hydroxycamptothecin, a topoisomerase I inhibitor. *Differentiation* **46**: 135–141

Liu LF and Miller KG (1981) Eukaryotic DNA topoisomerases: two forms of type I DNA topoisomerases from HeLa cell nuclei. *Proc Natl Acad Sci USA* **78**: 3487–3491

Liu LF and D'Arpa P (1992) Topoisomerase-targeting antitumor drugs: mechanisms of cytotoxicity and resistance. *Important Adv Oncol* **442**: 79–89

Lynch TJ, Kalish L, Strauss G, Elias A, Skariv A, Schulman LN, Posner M and Frei E (1994) Phase II study of topotecan in metastatic non-small cell lung cancer. *J Clin Oncol* **12**: 347–352

Ma J, Maliepaard M, Nooter K, Loos WJ, Kolker HJ, Verweij J, Stoter G and Schellens JHM (1996) Reduced cellular accumulation of topotecan, a novel mechanism of resistance in a human ovarian cancer cell line (to be submitted)

Malmstrom H, Sorbe B and Simonsen E (1996) The effect of topotecan in platinum refractory ovarian cancer. *Proc Am Soc Clin Oncol* **15**: 299

Moertel CCG, Schutt AJ, Reitemeier RJ and Hatin RG (1972) Phase II study of camptothecin (NSC 100880) in the treatment of advanced gastrointestinal cancer. *Cancer Chemother Rep* **56**: 95–101

Muggia FM, Creaven PJ, Hansen HH, Cohen MH and Selanrig OS (1972) Phase I clinical trial of weekly and daily treatment with camptothecin (NSC 100880): correlation with preclinical studies. *Cancer Chemother Rep* **56**: 515–521

Nakai H, Fukuoka M, Furuse K, Nakao I, Yoshimoni K, Ogura T, Hara N, Sakata Y, Saito H and Hasegawa K (1991) An early phase II study of CPT-11 for primary lung cancer. *Jpn Cancer Chemother* **18**: 607–612

Negoro S, Fukuoka M, Nitani H, Suzuki A, Nakabyashi T, Kimuru M, Motomiya M, Kupita Y, Hasegawa K and Kuriyama T (1991) An early phase II study of CPT-11, a camptothecin derivative, in patients with primary lung cancer. *Jpn J Cancer Chemother* **18**: 1013–1019

Nitiss J and Wang JC (1988) DNA topoisomerase-targeting antitumor drugs can be studied in yeast. *Proc Natl Acad Sci USA* **85**: 7501–7505

Noda K, Sasaki H, Yamamoto K, Yamamoto T, Hishimura R, Sugiyama T and Nakajama H (1996) Phase II trial of topotecan for cervical cancer of the uterus. *Proc Am Soc Clin Oncol* **15**: 280

Ohe Y, Sasaki Y, Sinkai T, Eguchi K, Tamura T, Kojima A, Kunikane H, Okamoto H, Karato A, Ohmatsu H, Kanzawa F and Saijo N (1992) Phase I study and pharmacokinetics of CPT-11 with 5-day continuous infusion. *J Natl Cancer Inst* **84**: 972–974

Ohno R, Okada K, Masaoka T, Kuramoto A, Arima T, Yoshida Y, Ariyoshi H, Ichimaru M, Sakai Y, Oguro M, Iti Y, Morishima Y, Yokomaku S and Ota K (1990) An early phase II study of CPT-11: a new derivative of camptothecin, for the treatment of leukemia and lymphoma. *J Clin Oncol* **8**: 1907–1912

Pantazis P, Hinz HR, Mendoza JT, Kozielski AJ, Williams LJ, Stehlin JS and Giovanella BC (1992) Complete inhibition of growth followed by death of human malignant xenografts in immunodeficient mice induced by camptothecins. *Cancer Res* **52**: 3980–3987

Pantazis P, Early JA, Kozielski AJ, Mendoza JT, Hinz HR and Glovanella BC (1993*a*) Regression of human breast carcinoma tumors in immunodeficient mice treated with 9-nitro-camptothecin: differential response of nontumorigenic and tumorigenic human breast cancer cells in vitro. *Cancer Res* **53**: 1577–1582

Pantazis P, Kozielski AJ, Vardeman DM, Petry ER and Giovanella BC (1993*b*) Efficacy of camptothecin congeners in the treatment of human breast carcinoma xenografts. *Oncol Res* **5**: 273–281

Pantazis P, Kozielski AJ, Mendoza JT, Early JA, Hinz HR and Giovanella BC (1993*c*) Camptothecin derivatives induce regression of human ovarian carcinomas grown in nude mice and distinguish between non tumorigenic and tumorigenic cells in vitro. *Int J Cancer* **53**: 863–871

Pantazis P, Harris N, Mendoza J and Giovanella B (1994*a*) Conversion of 9-nitro-camptothecin to 9-amino-camptothecin by human blood cells in vitro. *Eur J Hematol* **53**: 246–248

Pantazis P, Kozielski A, Rodriguez R, Petry E, Wani M, Wall M and Giovanella B (1994*b*) Therapeutic efficacy of camptothecin derivatives against human melanoma xenografts. *Melanoma Res* **4**: 5–10

Perez-Soler R, Fossella FV, Glisson BS, Lee JS, Murphy WK, Shin DM, Kemp BL, Lee JJ, Kane J, Robinson RA, Lippman SM, Kurie JM, Huber MH, Raber MN and Hong WK (1996) Phase II study of topotecan in patients with advanced

© Cancer Research Campaign 1997

non-small cell lung cancer (NSCLC) previously untreated with chemotherapy. *J Clin Oncol* **14**: 503–513

Phillips PC, Janss A, Kaufmann SH, Levow C, Yao Y and Colvin OM (1994) Topoisomerase I inhibitor schedule dependent activity and determinants of cytotoxicity in human brain tumors cell lines (abstract 2161). *Proc Am Assoc Cancer Res* **35**: 363

Plaxe S, Christen R, O'Quigley J, Braly P, Freddo J, McClay E, Heath D and Howell S (1993) Phase I trial of intra-peritoneal Topotecan (abstract 360). *Proc Am Soc Clin Oncol* **12**: 140

Potmesil M, Liebes L, Drygas J, Sehiya S, Morse L, Kozielski AJ, Wall ME, Wani MC, Stehlin JS and Giovanella BC (1995) Pharmacodynamics/pharmacokinetics of intragastric (IG) camptothecin analogs in a human cancer xenograft model (abstract 2652). *Proc Am Assoc Cancer Res* **36**: 445

Pratt CB, Steward C, Santana VM, Bowman L, Furman W, Ochs J, Marina N, Kuttesch JF, Heideman R, Sandlund J, Avery L and Meijer WH (1994) Phase I study of topotecan for pediatric patients with malignant solid tumors. *J Clin Oncol* **12**: 539–543

Puc HS, Bajorin DF, Bosl GJ, Amsterdam A and Motzer RJ (1995) Phase II trial of topotecan in patients with cisplatin-refractory germ cell tumors. *Investig New Drugs* **13**: 163–165

Recondo G, Abbruzzese J, Newman B, Newman R, Kuhn J, Von Hoff D, Garteiz D and Raber M (1991). A phase I trial of topotecan (TOPO) administered by a 24-hour infusion (abstract 1229). *Proc Assoc Cancer Res* **34**: 206

Reid JM, Burch PA, Benson LM, Gilbert JA, Richardson RL and Ames M (1992) Phase I clinical and pharmacologic evaluation of topotecan administered by a 24-hour continuous infusion (abstract 1553). *Proc Am Assoc Cancer Res* **33**: 259Robert F, Wheeler RH, Molthrop DC, Greene P and Chen S (1994) Phase II study of topotecan in advanced head and neck cancer: identification of an active new agent (abstract 905). *Proc Am Soc Clin Oncol* **13**: 281

Roca J (1995) The mechanisms of DNA topoisomerases. *Trends Biochem Sci* 156–160

Roethke S, Ozols RF and Hudes GR (1993) Phase II study of topotecan for hormone refractory prostate cancer (HRPC). *Proc Am Soc Clin Oncol* **12**: 247

Rothenberg ML, Rinaldi DA, Smith LS, Schaaf LJ, Hodges S, Thurman AM, Ichhpurani NK, Eckardt SG, Rodriguez GI, Villabona M, Drengle RR, Dietz AJ, Murphy TC, Burris MA and Von Mopp DD (1996) Every other week Trinotecan (CPT-II): Results of a phase I and pharmacokinetic (PK) study. *Proc Am Soc Clin Oncol* 15 (abstract 1561)

Rowinsky EK, Growchow LB, Hendricks CB, Ettinger DS, Forastiere AA, Hurowitz LA, McGuire WP, Sartorius SE, Lubejko BG, Kaufman SH and Donehower RC (1992) Phase I and pharmacologic study of topotecan: a novel topoisomerase I inhibitor. *J Clin Oncol* **10**: 647–656

Rubin E, Wood V, Bahrti A, Trites D, Lynch C and Kufe D (1994) A phase I trial of 9-amino-camptothecin (9AC) (abstract 1465). *Proc Am Assoc Cancer Res* **35**: 25

Sabiers JH, Berger NA, Berger SJ, Haaga JR, Hoppel CL and Wilson JKV (1993) Phase I trial of topotecan administration as a 72 hour infusion. *Proc Am Soc Cancer Res* **34**: 426

Saijo N, Nishio K, Kubota N, Kanzawa F, Shinkai T, Karato A, Ssaki Y, Eguchi K, Tamura T, Ohe Y, Oshita F and Nishio M (1994) 7-ethyl-10-[4-(1-piperidono)-1-piperidino] carbonyloxy camptothecin: mechanism of resistance and clinical trials. *Cancer Chemother Pharmacol* **34** (suppl.): 112–117

Saltz L, Sirott M, Young C, Tang W, Niedzweicki D, Tzy-Jyun Y, Tao Y, Trochanowski B, Wright P, Barbosa B, Toomasi F and Kelsen D (1992) Phase I and clinical pharmacologic study of intravenous topotecan alone and with granulocyte colony stimulating factor (G-CSF). *Ann Oncol* **3** (suppl. 1): 84

Samuels DS and Shimizu N (1992) DNA topoisomerase I phosphorylation in murine fibroblasts treated with 12-O-tetradecanoylphorbol-13 acetate and in vitro by protein kinase C. *J Biol Chem* **267**: 11156–11162

Schaeppi U, Fleischman RW and Cooney DA (1974) Toxicity of camptothecin (NSC-100880). *Cancer Chemother Rep* **5**: 25–36

Schellens JHM, Creemers GJ, Beynen JH, Rosing H, McDonald M, Davies B and Verweij J (1996) Bioavailability and pharmacokinetics of oral topotecan: a new topoisomerase I inhibitor. *Br J Cancer* **73**: 1268–1271

Schiller JH, Kim K and Johnson D (1994) Phase II study of topotecan in extensive stage small cell lung cancer. *Proc Am Soc Clin Oncol* **13**: 330

McSheehy PM, Gervasoni M, Lampasona V, Erba E and D'Incalci M (1991) Studies of the differentiation properties of camptothecin in human leukemia cells K562. *Eur J Cancer* **27**: 1406–1411

Smith RE, Lew D, Rodriguez GI, Taylos SA and Schuller D (1996) Evaluation of topotecan in recurrent or metastatic head and neck cancer. *Proc Am Soc Clin Oncol* **15**: 310

Stehlin JS, Natelson EA, Hinz HR, Giovanella BC, Ipolyi PD, Fehin KM, Trezona TP, Vardeman DM, Harris NJ, Marcee AK, Kozielski AJ and Ruiz-Razura A (1995) Phase I clinical trial and pharmacokinetic results with oral administration of 20-(S)-camptothecin. In *Campothecins: New Anticancer Agents*, Potmesil M and Pinedo H. (ed.), pp. 59–65. CRC Press: Boca Raton

Stewart AF and Schutz G (1987) Camptothecin-induced in vivo Topoisomerase I devagesin the transcriptionally active tyrosine aminotransferase gene. *Cell* **50**: 1109–1117

Subramanian D, Kraut E, Staubus A, Young DC and Muller MT (1995) Analysis of topoisomerase I/DNA complexes in patients administered topotecan. *Cancer Res* **55**: 2097–2103

Sugarman SM, Agani JA, Daugherty K, Winn R, Lanzotti V, Bearden JD and Abbruzzese JL (1994) A phase II trial of topotecan (TPT) for the treatment of advanced measurable colorectal cancer (abstract 686). *Proc Am Soc Clin Oncol* **13**: 225

Sugimoto Y, Tsukahara S, Oh-Hara T, Isoe T and Tsuruo T (1990) Decreased expression of DNA topoisomerase I in camptothecin-resistant tumor cell lines as determined by a monoclonal antibody. *Cancer Res* **50**: 6925–6930

Takeda S, Shimazoe T, Sato K, Sigimoto Y, Tsurno T and Kono A (1992) Differential expression of DNA topoisomerase I gene between CPT-11 acquired- and native resistant human pancreatic tumor cell lines: detected by RNA/PCR based quantitation assay. *Biochem Biophys Res Commun* **184**: 618–625

Takeuchi S, Dobashi K, Fujimoto S, Tanaka K, Suzuki M, Terashima Y, Hasumi K, Akiya K, Negishi Y and Tamay T (1991*a*) A late phase II study of CPT-11 on uterine cervical cancer and ovarian cancer. *Jpn J Cancer Chemother* **18**: 1681–1689

Takeuchi S, Takamizawa H, Takeda Y, Ohkawa T, Tamaya T, Noda K, Sugawa T, Sekiba K, Yakushiji M and Taguchi T (1991*b*) An early phase II study of CPT-11 for gynecologic cancers. *Jpn J Cancer Chemother* **18**: 579–584

Takimoto CH, Klecker RW, Dahut WL, Brillhart N, Yee LK, Strong JM, Nakashima H, Lieberman R, Allegra CJ and Grem JL (1994) Preliminary pharmacokinetics of the active lactone form of 9-aminocamptothecin using a sensitive new HPL assay. *Proc Am Assoc Cancer Res* **35**: 242

Takimoto CH, Dahut W, Harold N, Morrison GB, Quinn MF, Callen E, Liang MD, Arbuck SG, Chen A, Hamilton JM, Allegra CJ, Sorensen JM and Grem JL (1996) A phase I trial of a prolonged infusion of 9-amino-camptothecin (9AC) in adult patients with solid tumors. *Proc Am Soc Clin Oncol* **15**: 488

Tanizawa A and Pommier Y (1992) Topoisomerase I alteration in a camptothecin-resistant cell line derived from Chinese hamster DC3F cells in culture. *Cancer Res* **52**: 1849–1854

Tsuda H, Takatsuki K, Ohno R, Masaoko T, Okada K, Shirakawa S, Ohashi Y, Ohta K and Taguchi T (1992) A late phase II trial of a potent topoisomerase I inhibitor, CPT-11, in malignant lymphoma. *Proc Am Soc Clin Oncol* **11**: 316

Venditti JM (1971) Treatment schedule dependency of experimentally active antileukemic (L1210) drugs. *Cancer Chemother Rep* **2**: 35–59

Verschraegen CF, Natelson E, Giovanella B, Kavanagh JJ, Freedman RS, Kudelka AP, Edwards CL and Stehlin J (1996) Phase I study of oral 9-nitro-camptothecin. *Proc Am Soc Clin Oncol* **15**: 482

Verweij J, Lund B, Beynen J, Planting A, De Boer-Dennert M, Koier I, Rosing H and Hansen H (1993) Phase I and pharmacokinetics study of topotecan, a new topoisomerase I inhibitor. *Ann Oncol* **4**: 673–678

Wall ME, Wani MC, Cook CE, Palmer KH, McPhail AT and Lim GA (1966) Plant antitumor agents. I. The isolation and structure of camptothecin, a novel alkaloid leukemia and tumor inhibitor from camptotheca acuminata. *J Am Chem Soc* **88**: 3888–3890

Wanders J, Ardizanni A, Hansen HH, Dombernowsky P, Postmus PE, Buitenhuis M, McDonald M, Giaccone G and Verweij J (1995) Phase II study of topotecan in refractory and sensitive small cell lung cancer (SCLC) (abstract 1415). *Proc Am Assoc Cancer Res* **36**: 237

Wanders J, Ten Bokkel Huinink WW, Heinrich B, Gore M, Calvert AH, Lehnert M, Ten Velde A and Verweij J (1996) Phase II studies with GI147211 in 5 different tumor types. Preliminary results. NCI-EORTC symposium on new drugs in cancer therapy. Amsterdam, March, p.131

Zhang H, Wang JC and Liu LF (1988) Involvement of DNA topoisomerase I in transcription of human ribosomal RNA genes. *Proc Natl Acad Sci USA* **85**: 1060–1064

© Cancer Research Campaign 1997